# Proximal labeling of the Golgi secretome reveals fat body–derived humoral factors in *Drosophila* disc regeneration

**DOI:** 10.1016/j.jbc.2026.113198

**Published:** 2026-05-27

**Authors:** Yutaka Yoshida, Soshiro Kashio, Masayuki Miura

**Affiliations:** 1 Department of Genetics, Graduate School of Pharmaceutical Sciences, The University of Tokyo, Tokyo, Japan; 2 Ubiquitin Biology Laboratory, Graduate School of Frontier Biosciences, The University of Osaka, Osaka, Japan; 3 Laboratory for Cell Vigor Regulation, National Institute for Basic Biology, Okazaki, Aichi, Japan; 4 Basic Biology Program, The Graduate University for Advanced Studies, SOKENDAI, Okazaki, Aichi, Japan

**Keywords:** Golgi, N-glycosylation, biotin labeling, proteomics, secreted protein

## Abstract

Humoral factors act as inter-tissue mediators and regulate various organismal physiologies. Although the importance of humoral factors in tissue repair has been recently recognized, our understanding of how humoral proteins regulate tissue repair remains limited. Glycosylation is an important modification of the conventional secretory pathway, and we have demonstrated that N-glycosylation in the Golgi apparatus of the *Drosophila* fat body, the major secretory tissue equivalent to the mammalian liver and adipose tissue, remotely contributes to epithelial tissue repair. To identify humoral factors that contribute to repair *via* the Golgi apparatus, we constructed a Golgi-specific protein biotinylation system and performed hemolymph proteomics. By combining genetic analyses, we found that fat body–derived innate immune regulators and iron mediators affect tissue repair. Altogether, our Golgi-specific labeling system has the potential to identify Golgi-mediated secreted factors that regulate inter-organ communication.

In multicellular organisms, the functions of each organ are highly specialized and coordinated. Various tools for analyzing inter-organ communication have recently emerged. In *in vivo* experimental systems, fluorescent-labeled exosomes are expressed in an organ-specific manner, and inter-organ communication was analyzed using zebrafish embryos (1). A method has also been developed to identify the trafficking of secreted proteins by protein biotin labeling in the endoplasmic reticulum (ER) (2).

Tissue regeneration is a homeostatic mechanism against tissue loss that is observed in specific organs and animals (3, 4). Nonautonomous tissue regulation of repair has also been examined. For example, the vagus–macrophage axis regulates liver regeneration in mice, and platelet-derived serotonin mediates liver regeneration (5, 6). *In vitro* screening showed that myristoylated alanine-rich C kinase substrate–like protein, a factor secreted from the wound epidermis, is required for the initial cell cycle response during axolotl appendage regeneration (7). Although the mechanisms of regenerative regulation through inter-organ communication have been identified, a comprehensive analysis of secreted proteins contributing to tissue regeneration has not yet progressed at the organismal level. *Drosophila* is one of the model organisms that contributes to the systemic aspects of various phenomena through tissue-specific genetic manipulation. Additionally, imaginal discs in *Drosophila* larvae, the epithelial precursors of adult tissues, have been used as models of tissue regeneration (8). In our previous study, we revealed the contribution of the fat body, a counterpart of the mammalian liver and adipose tissue, to wing disc repair (9, 10, 11). We found that kynurenic acid, a humoral metabolite secreted by the fat body, was remotely regulated by tachykinin neurons and was necessary for imaginal disc repair (9, 12). Considering that the fat body is a major endocrine organ in flies, we have focused on the fat body as a source of humoral proteins regulating disc regeneration.

In this study, we attempted to identify secretory proteins that contribute to tissue regeneration. Most proteins are secreted *via* the ER–Golgi pathway, referred to as the conventional secretory pathway, and undergo glycosylation in the ER lumen or Golgi apparatus (13, 14). Glycosylation is important for the functioning of secreted factors; however, our understanding of its contribution to tissue regeneration is limited. Therefore, we tested whether glycosyltransferases and sugar hydrolases contribute to wing disc regeneration and found that the RNAi knockdown of glycosyltransferases and sugar hydrolases in the fat body hampered wing disc repair without any obvious effect on development. Using this finding as a starting point, we conducted a comprehensive search for secreted proteins that contribute to regeneration, using Golgi-derived secretome analyses and RNAi screening.

## Results

### N-glycosylation pathway in the ER and Golgi apparatus contributes to disc regeneration

**Figure 1:**
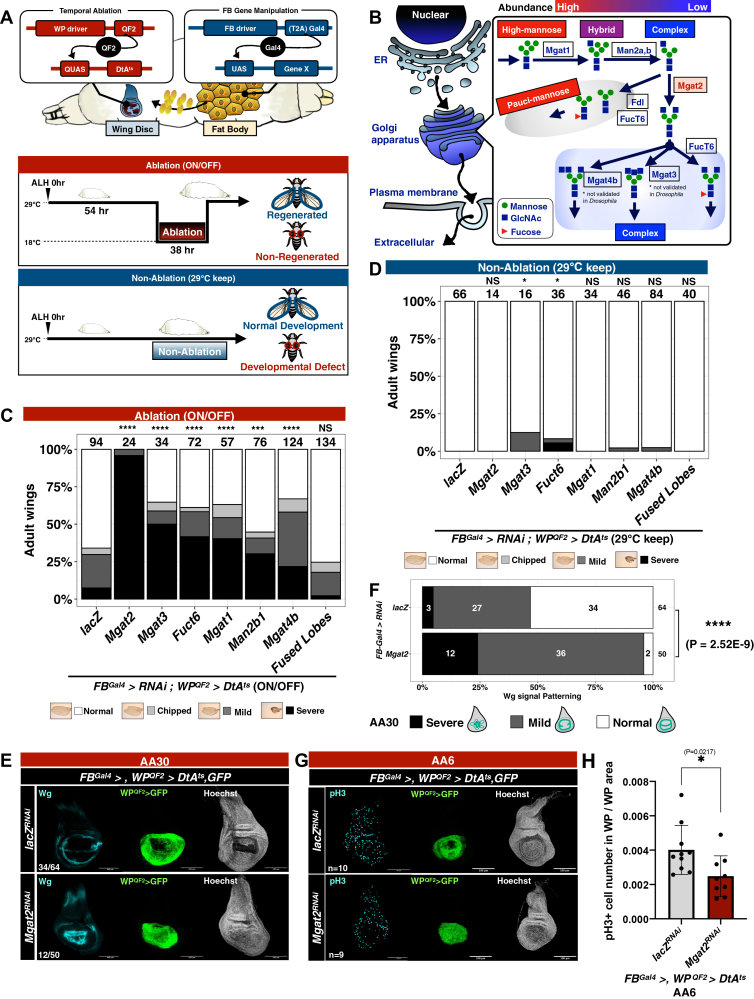
**Glycosyltransferases and sugar hydrolases in fat body for disc regeneration.***A*, schematic view of temporal disc ablation and gene manipulation in fat body. Temporal disc damage is induced by DtA^ts^ at a low temperature (18 °C) for 38 h and returned to 29 °C after ablation. This condition is indicated as “ablation.” In a nondamaged condition, temperature shift is not induced and kept at 29 °C to avoid damage induction by DtA^ts^. This condition is indicated as “nonablation.” *B*, N-glycosylation pathway in the Golgi apparatus. It is important to note that Mgat1 and Mgat2 were functionally validated in *Drosophila*, but Mgat3 and Mgat4b were not. The *Drosophila* N-glycans are dominated by high-mannose and paucimannose-type glycans, whereas GlcNAc-extended antennae are found predominantly in hybrid-type structures and only to a lesser extent in complex-type glycans. Fdl is expected to act on substrates regardless of core fucosylation status by FucT6. *C* and *D*, comparison of adult wing sizes between ablation (*C*) and nonablation (*D*). RNAi knockdown of N-glycosylation enzymes hampered disc regeneration. RNAi knockdown was induced in fat body by *FB*^*Gal4*^. Statistical analysis was conducted using Fisher’s exact test to compare control (*WP*^*QF2*^*> DtA*^*ts*^*; FB*^*Gal4*^*> lacZ*^*RNAi*^) with treated larvae. NS, not significant, ∗∗: *p* < 0.01, ∗∗∗∗: *p* < 0.0001. Number of flies is listed above each genotype in the bar graph. *E* and *F*, representative examples of wing discs developed within the indicated time course. Discs were dissected at 30 h after ablation (AA30). Fat body–specific RNAi knockdown of *Mgat2* affected Wg patterning in disc regeneration. Discs were stained with anti-Wg antibody and Hoechst33342. The white scale bar represents 100 μm. Abnormality of Wg patterning in wing pouch was classified into 3 groups, and classification was quantified in (*F*). Fisher’s exact test was applied. ∗∗∗∗: *p* < 0.0001. Number of wing discs is listed in image or next to each genotype in the bar chart. *G* and *H*, representative examples of wing discs developed within indicated time course. Discs were dissected at 6 h after ablation (AA6). Fat body–specific RNAi knockdown of *Mgat2* affected cell proliferation in disc regeneration. Discs were stained with anti-pH3 antibody and Hoechst33342. The white scale bar represents 100 μm. Normalized pH3-positive cell numbers in wing pouch region were quantified in (*H*). Error bars indicate standard error of the mean. An unpaired *t* test was applied. ∗: *p* < 0.05. Number of wing discs is listed in (*G*). DtA^ts^, temperature-sensitive diphtheria toxin A domain; Fdl, fused lobes; pH3, phospho-histone 3; Wg, wingless.

To induce temporal tissue damage and subsequent wing disc regeneration, a temperature-sensitive diphtheria toxin A domain (DtA^ts^) was used for temporal cell ablation and repair in our previous study (10) (Fig. 1*A*). Briefly, larvae were reared at 29 °C to stop cell ablation and temporally reared at 18 °C to induce cell ablation. After hatching, we examined the regenerating ability by observing the adult wing phenotype. To manipulate Gal4/UAS-mediated gene expression in the fat body, DtA^ts^ was independently induced by the Q system, another binary system, in the wing pouch (WP) region of the wing disc, which becomes an adult wing through metamorphosis. We also examined the effects of genetic manipulation on development in the fat body by maintaining the temperature at 29 °C. Using this system, we attempted to identify regenerative regulatory proteins secreted from the fat body.

First, we examined the effects of general secretion from the fat body on disc regeneration. A previous study showed that factors involved in the protein transport pathway contribute to general secretion from the fat body (15). RNAi knockdown of several factors involved in the secretory pathway, such as *Rab1*, *Syx18*, and *Rab11*, and endocytosis, such as *AP2α* and *Shibire*, caused a lethal phenotype, indicating their essential role in the fat body during development (Fig. S1, *A*–*C*). In contrast, RNAi knockdown of other factors involved in secretion and endocytosis, such as *Sec23*, *deltaCop*, and *Tweek*, increased the percentage of the worsened wing phenotype (Fig. S1*B*). RNAi knockdown of *Sec23*, *deltaCOP*, and *Tweek*, which causes regeneration failure, had little effect on normal wing formation (Fig. S1*C*). These results suggest that protein secretion from the fat body to wing discs during ablation supports wing disc regeneration, whereas impairment of several protein transport regulators causes lethality.

When humoral proteins are secreted *via* the conventional secretory pathway, most of them undergo a series of glycosylation steps as they move from the ER to the Golgi apparatus (14). Focusing on the glycosylation process as a characteristic of secretion, rather than the secretory pathway itself, could facilitate screening with a minimal impact on development. Therefore, we investigated whether glycosyltransferases and sugar hydrolases contributed to disc regeneration. N-glycosylation in *Drosophila* is classified into high-mannose, pauci-mannose, complex, and hybrid types. Notably, the *Drosophila* N-glycans are dominated by high-mannose and paucimannose-type glycans, whereas GlcNAc-extended antennae are found predominantly in hybrid-type structures and only to a lesser extent in complex-type glycans (16, 17) (Fig. 1*B*). We examined the roles of the enzymes before and after branching of the N-glycosylation pathway (Fig. 1*B*). We observed that fat body–specific RNAi knockdown of the enzyme, *fused lobes*, specifically contributing to the pauci-mannose pathway, did not affect disc repair. However, fat body–specific RNAi knockdown of enzymes contributing complex and hybrid N-glycosylation pathway resulted in a worsened wing phenotype after disc damage (Fig. 1*C*). Furthermore, RNAi knockdown of ER glycosyltransferases (*Alg3* and *Alg9*), the ER glycosidase *GCS1*, and Golgi glycosidases involved in the early N-glycosyl modification (*Man1b,* and *Man1c*) also caused regenerative inhibition (Fig. S1, *D* and *E*). Fat body–specific RNAi knockdown of glycosyltransferases and sugar hydrolases during normal development had no significant effect on wing formation (Figs. 1*D* and S1*F*). Among the glycosyl-modifying enzymes examined, we focused on Mgat2 as it acts on the complex N-glycosylation branch and significantly affects disc-regeneration phenotype in the DtA^ts^ model (Fig. 1, *B* and *C*). Although the RNAi knockdown efficiencies of *Mgat1* and *Mgat2* were found to be similar (Fig. S2, *A* and *B*), we refrain from directly comparing the severity of their phenotypes due to potential differences in RNAi efficacy and downstream sensitivity. Notably, residual *Mgat1* expression was detected after knockdown (Fig. S2*B*), which may be sufficient to support downstream glycan processing given the abundance of high-mannose–type glycans in *Drosophila* (17).

Given that N-glycosylation can influence protein folding, stability, and secretion (13, 14), we then investigated whether the perturbation of N-glycan processing in the fat body affects the abundance of a representative secreted factor. We focused on Eiger (Egr)/TNF, an N-glycosylated protein produced by fat body (18). Upon fat body-specific *Mgat1* RNAi knockdown, the secreted form of Egr protein levels tended to decrease (Fig. S2, *C* and *C*′). In contrast, the fat body–specific *Mgat2* RNAi knockdown resulted in a marked increase in Egr protein levels (Fig. S2, *C* and *C*′). Consistent with previous reports (18), Egr exhibited a mobility shift upon PNGase F treatment, confirming that fat body–derived Egr was N-glycosylated (Fig. S2*D*). These results suggest that N-glycan processing contributes to the steady-state levels of the secreted form of Egr protein, with Mgat2-dependent steps exerting an impact under our experimental conditions.

To validate the tissue specificity of the fat body drivers used in this study, we examined the expression patterns of *C10*^*Gal4*^, *FB*^*Gal4*^, and *Gnmt*^*T2A-Gal4*^. Whole-larval imaging of the *UAS-GFP* reporter showed that *C10*^*Gal4*^ drove broad expression, including in the wing disc and brain, as well as in the salivary gland, whereas *FB*^*Gal4*^ expression was predominantly detected in the fat body with an additional signal in the salivary gland (Fig. S2*E*). In contrast, *Gnmt*^*T2A-Gal4*^ expression was restricted to the fat body (Fig. S2*E*). We further assessed whether these fat body drivers exhibited unintended expression in the wing disc, using the *UAS-RedStinger* reporter at two time points (6 h after injury; AA6, and 6 h after nonablation [NA]; NA6). As expected, *rn*^*Gal4*^ was strongly expressed in the WP at both time points, whereas *FB*^*Gal4*^ and *Gnmt*^*T2A-Gal4*^ showed little or no detectable expression in the wing disc under either condition (Fig. S2, *F* and *F*′). Together, these results indicated that *FB*^*Gal4*^ and *Gnmt*^*T2A-Gal4*^ functioned as fat body–specific drivers in our experimental settings.

Ablation by DtA^ts^ caused a developmental delay, as previously described (10), but RNAi knockdown of *Mgat2* did not affect developmental speed (Fig. S3*A*), indicating that Mgat2 regulates disc repair by affecting repair processes. Gene expression of glycosyltransferases and sugar hydrolases, except for the moderately expressed *Mgat4b*, at 0 h after injury (after ablation [AA] at 0 h, AA0) and 6 h (AA6), showed no significant change between ablation and NA conditions (Fig. S4, *A*–*E*).

Next, we investigated whether Mgat2 in the fat body contributed to wing disc remodeling after injury by observing the expression of the morphogen wingless (Wg) and GFP-labeled WP regions (*WP*^*QF2*^*>mCD8-GFP*). RNAi knockdown of *Mgat2* in the fat body did not significantly affect the WP region area recovery but affected Wg repatterning at 30 h after injury (AA30), a late stage of repair (Figs. 1, *E*, *F* and S3, *B*, *C*). This Wg patterning abnormality was not due to excessive cell death by staining for cleaved Dcp1, a marker for activated caspase (Fig. S3, *B* and *D*). Although the recovery area was not affected at AA30, the number of mitotic cells (phospho-histone 3 [pH3]-positive cells) in the early stage of regeneration (AA6) was reduced upon the RNAi knockdown of *Mgat2* in the fat body (Fig. 1, *G* and *H*). Consequently, Mgat2 in the fat body is required to restore cell proliferation during the early stages of repair.

### Constructing a system for biotin labeling in the Golgi apparatus

**Figure 2:**
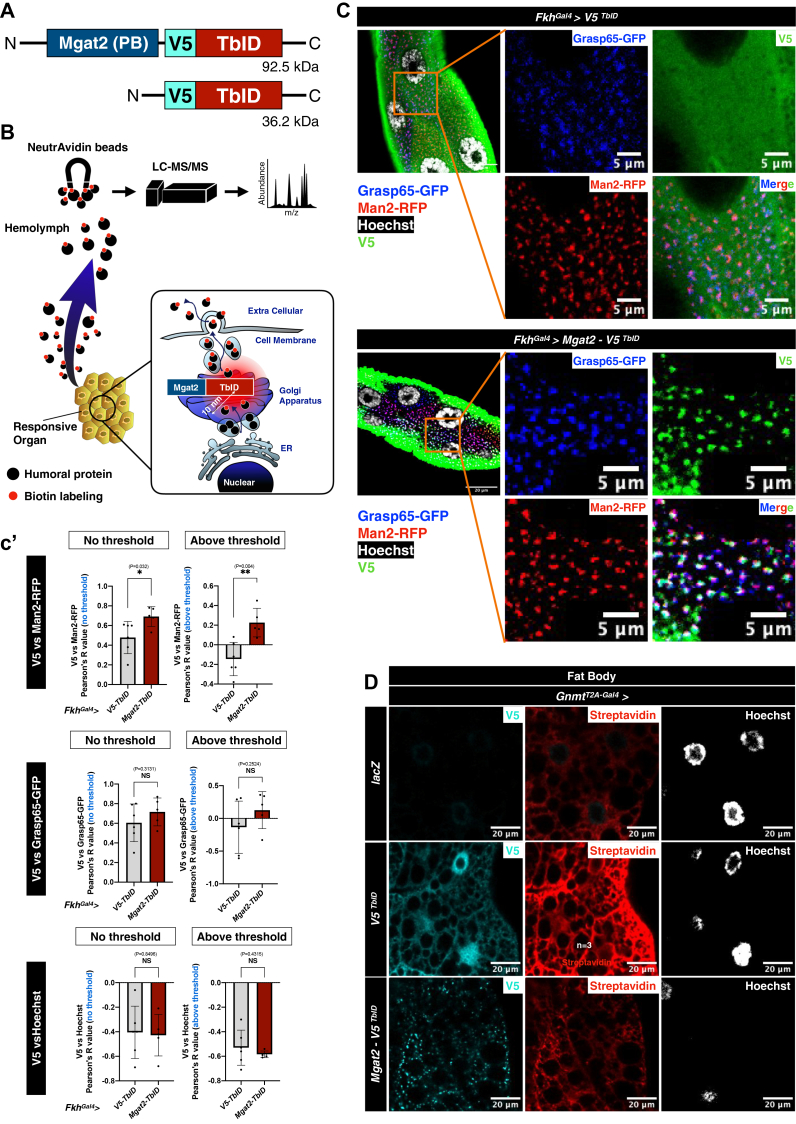
**Subcellular localization and biotin-labeling of Mgat2-TbID.***A*, diagram of V5-TbID and Mgat2-TbID (isoform PB) constructs. *B*, schematic view of secretome analysis. Tissue-specific expression of *Mgat2-TbID* enables comprehensive biotin-labeling of secretory pathway proteins *via* near-labeling in the Golgi apparatus. *C*, cellular localization of Mgat2-TbID and V5-TbID. Overexpression of each genotype in salivary gland with *Fkh*^*Gal4*^. V5 signal (*green*) shows localization of each construct. Grasp65 (*blue*) is a marker of *cis*-Golgi, and Man2 (*red*) is a marker of medial-Golgi. The white scale bar represents 5 or 20 μm. Number of samples was 5 for *Fkh*^*Gal4*^*> V5*^*TbID*^ and 6 for *Fkh*^*Gal4*^*>Mgat2-V5*^*TbID*^. *C′,* calculation of Pearson’s correlation coefficients (no_threshold and above_threshold) between the V5 signal and two Golgi markers, Man2-RFP and Grasp65-GFP, and between the V5 signal and Hoechst, using the Coloc 2 plugin in Fiji software. The number of samples was 5 for *Fkh*^*Gal4*^*> V5*^*TbID*^ and 6 for *Fkh*^*Gal4*^*>Mgat2- V5*^*TbID*^. Mgat2-TbID exhibited higher Pearson’s R values with Man2-RFP (mean no-threshold R = 0.69; mean above-threshold R = 0.23) than V5-TbID (mean no-threshold R = 0.48; mean above-threshold R = −0.15). Mgat2-TbID also showed higher correlation with Grasp65-GFP (mean no-threshold R = 0.72; mean above-threshold R = 0.13) than V5-TbID (mean no-threshold R = 0.61; mean above-threshold R = −0.14). *D*, subcellular localization and biotin-labeling of Mgat2-TbID and V5-TbID. Overexpression of each genotype in fat body with *Gnmt*^*T2A-Gal4*^. V5 signal (*cyan*), biotin-labeling (*red*), and nuclear (*gray*) were stained with anti-V5 antibody, Streptavidin-Cy3, and Hoechst33342, respectively. The white scale bar represents 20 μm. Number of samples was n = 10 in *Gnmt*^*T2A-Gal4*^*>lacZ*, n = 10 in *Gnmt*^*T2A-Gal4*^*> V5*^*TbID*^ and n = 6 in *Gnmt*^*T2A-Gal4*^*>Mgat2- V5*^*TbID*^.

To identify regenerative proteins secreted from the fat body, we aimed to comprehensively detect secretory proteins using TurboID (hereafter referred to as TbID), a tool that biotinylates proximal proteins within a 10 nm range (19). In a previous study, BirA∗G3, an earlier version of TbID, was expressed in the *Drosophila* ER lumen in a tissue-specific manner, enabling the detection of secretory proteins in the specific tissues of other organs (2). In our study, we focused on the Golgi apparatus because complex N-glycosylation was found to affect disc regeneration (Fig. 1, *B*–*D*). We analyzed tissue-specific secretory proteins by fusing TbID with a glycosyltransferase present in the Golgi apparatus. Given that fat body–specific RNAi knockdown of Mgat2 produced a regeneration failure among the conditions tested (Fig. 1*C*), and because Mgat2 acts upstream of the complex-type N-glycosylation branch, we generated *Mgat2-TbID* transgenic flies by fusing the C terminus of Mgat2 with a V5 tag and TbID (Fig. 2, *A* and *B*).

When V5-TbID was expressed in the salivary gland using *Fkh*^*Gal4*^ for observation, the V5 signal diffused within the cell, whereas the Golgi membrane markers Grasp65-GFP (*cis*-Golgi) and Man2-RFP (medial Golgi) formed puncta (Fig. 2*C*) (20). In contrast, the expression of Mgat2-V5-TbID (predicted to localize to the Medial Golgi (21), hereafter referred to as Mgat2-TbID) in the salivary gland resulted in puncta formation, and the puncta of Mgat2-TbID localized in close proximity to Golgi membrane markers (Fig. 2*C*). To quantitatively assess the extent of colocalization, we performed Pearson’s correlation analysis for V5 with Man2-RFP (medial-Golgi marker), Grasp65-GFP (*cis*-Golgi marker), and Hoechst (Fig. 2*C*’), using both whole-image (no-threshold) and above-threshold (the Costes method) Pearson’s correlation coefficients to capture the global signal distribution and enrichment of high-intensity structures. Consistent with its predicted Golgi localization, Mgat2-TbID showed higher Pearson’s R values with Man2-RFP (mean no-threshold R = 0.69, above-threshold R = 0.23) compared with V5-TbID (mean no-threshold R = 0.48, above-threshold R = −0.15). Mgat2-TbID also exhibited a higher Pearson’s R value in comparison with Grasp65-GFP (mean no-threshold R = 0.72, above-threshold R = 0.13), than V5-TbID (mean no-threshold R = 0.61, above-threshold R = −0.14), indicating preferential association with Golgi structures. In contrast, both Mgat2-TbID and V5-TbID displayed negative Pearson’s R values when compared with Hoechst staining (Fig. 2*C*′), demonstrating that neither construct colocalized with the nucleus.

Furthermore, we investigated the signal of biotin-labeled proteins and V5 tags in the fat body. Unlike lacZ (control), V5-TbID was detected in both Hoechst-positive (nuclear) and Hoechst-negative regions of the cell, suggesting a broad intracellular distribution (Fig. 2*D*). Notably, the signal appeared to be excluded from certain regions within the cell, which may correspond to lipid droplet-rich areas characteristic of the fat body (Fig. 2*D*). Biotin-labeled proteins marked with streptavidin were detected primarily in the cytoplasm. For Mgat2-TbID, punctate signals of V5 and biotin-labeled proteins were detected in the cytoplasm (Fig. 2*D*). Streptavidin staining showed a broader distribution and did not fully recapitulate the punctate V5 pattern (Fig. 2*D*). This is likely due in part to background staining from endogenous biotin or biotinylated proteins, as also observed in the lacZ control, which reduces the contrast of discrete structures. Given that the fat body functions as a major nutrient storage organ, basal levels of endogenous biotin and biotinylated proteins are expected and likely account for the background streptavidin staining observed in the lacZ control. Nevertheless, compared to the lacZ control, the Mgat2-TbID sample displayed increased signal intensity and a distinct spatial distribution, indicating TbID-dependent biotinylation rather than endogenous background. Additionally, the broader distribution of streptavidin signal relative to V5 may reflect the trafficking of biotinylated cargo proteins beyond the Golgi. Together, these results support the interpretation that Mgat2-TbID localizes to the Golgi apparatus and preferentially labels proteins associated with the secretory pathway, rather than causing nonspecific cytosolic biotinylation.

### Analysis of secreted proteins from the fat body using Mgat2-TbID identified a group of proteins responsible for regeneration

**Figure 3:**
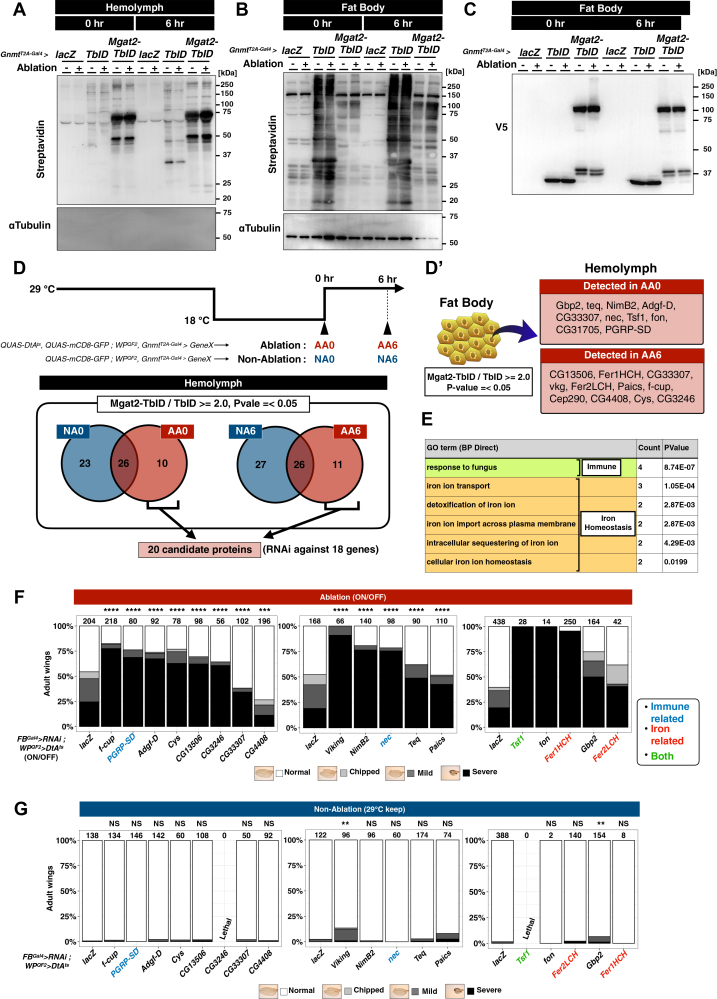
**Secretome analysis identified fat body–derived iron- and immune-related proteins are necessary for disc regeneration.***A* and *B*, Western blotting for biotinylated proteins (detected by Streptavidin-HRP)in hemolymph (*A*) and fat body (*B*). *LacZ* (Control without TbID), V5-TbID, and *Mgat2-*TbID were expressed in fat body with *Gnmt*^*T2A-Gal4*^. Samples were collected both in ablation and nonablation conditions and at 0 and 6 h time points. α-Tubulin was used as an internal control. *C*, Western blotting for V5-tagged proteins in fat body. V5-TbID and Mgat2-TbID were estimated to be 36.2 kDa and 92.5 kDa, respectively. *D* and *D′,* scheme for temperature change and sampling. The analysis of biotinylated proteins in hemolymph and fat body at two time points. Under each condition, proteins detected with *Mgat2-*TbID at more than twice the amount detected with *V5-*TbID (*p* < 0.05) were extracted. CG33307 in hemolymph overlapped in both AA0 and AA6. Biological replicates were n = 3. *E*, GO analysis of 20 proteins in (*D*) with DAVID. *F and G*, RNAi screening of candidate proteins in ablation (*F*) and nonablation (*G*). Immune-related proteins are indicated in *blue*, iron-related proteins are indicated in *orange*, and proteins involved in both are indicated in *magenta*. Statistical analysis was conducted using Fisher’s exact test to compare control (*WP*^*QF2*^*> DtA*^*ts*^*; FB*^*Gal4*^*> lacZ*^*RNAi*^) with treated larvae. NS: not significant, ∗∗: *p* < 0.01, ∗∗∗: *p* < 0.001, and ∗∗∗∗: *p* < 0.0001. Number of flies is listed above each genotype in the bar graph. AA, after ablation; DAVID, Database for Annotation, Visualization, and Integrated Discovery; DtA^ts^, temperature-sensitive diphtheria toxin A domain; GO, gene ontology; HRP, horseradish peroxidase.

To validate whether Mgat2-TbID can label secretory proteins, we examined Mgat2-TbID expression in the fat body. In order to select suitable fat body–specific Gal4 drivers for both V5-TbID and Mgat2-TbID expression, we examined three fat body–Gal4 drivers (*FB*^*Gal4*^, *Gnmt*^*T2A-Gal4*^, and *r4*^*Gal4*^). Both V5-TbID and Mgat2-TbID in the fat body were observed with *Gnmt*^*T2A-Gal4*^ and *r4*^*Gal4*^ but not with FB^Gal4^ (Fig. S5*A*). Consistently, biotin-labeled proteins, detected by streptavidin in both the hemolymph and fat body, were present in *Gnmt*^*T2A-Gal4*^ and *r4*^*Gal4*^ samples but absent in those from FB^Gal4^ (Fig. S5, *A* and *B*). These results therefore indicate that both V5-TbID and Mgat2-TbID can be effectively expressed using *Gnmt*^*T2A-Gal4*^ and *r4*^*Gal4*^, although V5-TbID can only be expressed using *FB*^*Gal4*^. Given these findings and the technical difficulty of genetic recombination of *r4*^*Gal4*^ and *WP*^*QF2*^, we selected *Gnmt*^*T2A-Gal4*^ for subsequent Mgat2-TbID analyses. Western blot analysis of biotinylated proteins in disc regeneration showed that *Drosophila* wandering third instar larvae expressing Mgat2-TbID exhibited labeled proteins in both hemolymph and the fat body (Fig. 3, *A* and *B*). In the hemolymph, labeled Mgat2-TbID proteins were detected at much higher levels than lacZ and V5-TbID, and the banding patterns of Mgat2-TbID closely resembled those of BirA∗G3-ER (Fig. S5*C*). Cytosolic α-tubulin was not detected in the hemolymph samples. In fat body, V5-TbID strongly labeled cellular proteins (Fig. S5*D*). Additionally, Mgat2-TbID and BirA∗G3-ER showed a similar banding pattern in the hemolymph and fat body, implying that Mgat2-TbID efficiently labeled the proteins of secretory pathway (Fig. S5, *C* and *D*).

Although Mgat2-TbID appeared to efficiently label the proteins within the secretory pathway, the membrane-anchored Golgi glycosyltransferase was cleaved at the N terminus, and catalytic enzymes were secreted into the extracellular space (22). In order to determine whether V5-TbID or Mgat2-TbID itself is secreted, we investigated V5-TbID and Mgat2-TbID secretion by expressing them in the fat body using *Gnmt*^*T2A-Gal4*^ and *r4*^*Gal4*^, and found their presence in the hemolymph (Fig. S5, *B* and *C*). However, since TbID functions in an ATP-dependent manner and extracellular ATP levels are extremely low in the human plasma (23), it can be speculated that TbID does not function well in the hemolymph. To directly test whether secreted TbID retains catalytic activity in the hemolymph and could not drive off-tissue labeling, we performed an *ex vivo* hemolymph incubation assay. Hemolymph collected from *Gnmt*^*T2A-Gal4*^*>V5-TbID* or *Gnmt*^*T2A-Gal4*^*>Mgat2-TbID* larvae reared on standard food was incubated with 50 μM biotin for 1 h in the presence or absence of supplemented 100 μM ATP. ATP addition did not increase the overall level of hemolymph protein biotinylation in either condition (Fig. S5*E*). If secreted TbID were enzymatically active in the extracellular hemolymph environment, ATP supplementation would be expected to enhance biotinylation and to diminish this consistent difference between V5-TbID and Mgat2-TbID, which was not observed. This suggests that either the amount of secreted TbID is low or that its enzymatic activity is not fully expressed. These results also indicate that the biotinylated proteins detected in the hemolymph were predominantly labeled *in vivo* prior to collection rather than newly labeled during the *ex vivo* incubation.

Additionally, we have expressed *V5-TbID* and *Mgat2-TbID* in several cell types and tissues using *nSyb*^*Gal4*^ for neurons, *Pros*^*Gal4*^ for neural cells and gut enteroendocrine cells, *Mef2*^*Gal4*^ for muscle, *Pxn*^*Gal4*^ for hemocytes, and *Fkh*^*Gal4*^ for the salivary glands (Fig. S5, *B*, *F* and *G*). Labeled proteins were detected in the hemolymph by expressing *V5-TbID* and *Mgat2-TbID* using *Mef2*^*Gal4*^ and *Pxn*^*Gal4*^. Therefore, *V5-TbID* and *Mgat2-TbID* can be used in several cells and tissues other than fat body.

To identify proteins specifically secreted from the fat body during regeneration, we conducted fat body–specific protein labeling and proteomics analysis of the hemolymph and fat body at the early stage of disc repair (AA0 and AA6) (Fig. 3*D*). Volcano plots of the Mgat2-TbID to V5-TbID ratios in the hemolymph proteomics dataset across all four experimental conditions (NA0, NA6, AA0, and AA6) provided a comprehensive overview of the proteome-wide changes associated with Mgat2-TbID–dependent biotinylation (Fig. S6, *A*–*D*). To further assess reproducibility, we analyzed replicate correlations by calculating the abundance ratios (Mgat2-TbID *versus* lacZ control) for each biological replicate (n = 3) and visualized these data in a 3D correlation plot (Fig. S6*E*). Proteins with high or low detection levels showed minimal deviation across replicates, indicating low technical variability and robust replicate-to-replicate agreement.

Western blot analysis indicated no significant difference in labeled proteins detected in the hemolymph or fat body at any time point between the ablation and NAgroups (Fig. 3, *A* and *B*). There were also no differences in the amounts of V5-TbID or Mgat2-TbID proteins present in the fat body, regardless of the time point or ablation (Fig. 3*C*). As seen in the Western blot analysis (see above), Mgat2-TbID and BirA∗G3-ER labeled similar numbers of proteins in both fat body proteomics (Fig. S7*A*) and hemolymph proteomics (Fig. S7*B*). Cellular component and pathway analyses were performed using the Database for Annotation, Visualization, and Integrated Discovery (DAVID) (https://david.ncifcrf.gov). Cellular component analysis indicated that most of the proteins labeled by V5-TbID were present in the cytosol and nucleus under all conditions (Table S1), consistent with the observation that V5-TbID expression was also observed in the cytosol and nucleus (Fig. 2, *C* and *D*). Membrane proteins were the top hits in both Mgat2-TbID and BirA∗G3-ER but not in V5-TbID in the fat body (Table S1). ER-related proteins were also more enriched in both BirA∗G3-ER and Mgat2-TbID, and proteins related to the Golgi apparatus were enriched in Mgat2-TbID under all conditions (Table S1). Furthermore, well-known major secreted proteins from the fat body, such as Lsp1α, γ, β, and Lsp2, were labeled more in the Mgat2-TbID samples when compared to those of V5-TbID samples in the hemolymph (Fig. S7*C*). These data indicate that secretory pathway proteins were more specifically labeled by Mgat2-TbID and BirA∗G3-ER than by V5-TbID. In the pathway analysis, N-glycosylation-related terms were more frequently detected in Mgat2-TbID than in others, whereas terms related to ER function were enriched in both BirA∗G3-ER and Mgat2-TbID (Table S2). In summary, these results suggest that the proteins detected in both Mgat2-TbID and BirA∗G3-ER were relatively similar, whereas Mgat2-TbID more specifically labeled secretory proteins within the Golgi apparatus.

To identify proteins secreted from the fat body into the hemolymph during regeneration, we compared the labeled proteins, which were two times higher in Mgat2-TbID than in V5-TbID (Fig. 3, *D*, *D′*, and Table S3). We identified 20 candidate regenerative secretory proteins from the fat body in AA0 and AA6 by focusing on the factors that were undetected in NA but detected only in after-ablation (AA) (Table S4). Gene ontology (GO) analysis of these regenerative candidate proteins using DAVID revealed the upregulation of factors related to innate immunity and iron homeostasis (Fig. 3*E*). In addition, we compared the labeled proteins, which were two times higher in BirA∗G3-ER than in V5-TbID (Fig. S7, *D*, *D′*, and Table S3). We identified 14 proteins as regenerative candidate proteins by focusing on the factors that were undetected in NA but detected only in AA. GO analysis of these proteins also revealed the upregulation of factors related to iron homeostasis and innate immunity (Fig. S7*E*). These results suggest that the factors contributing to the regeneration of wing discs are related to iron binding and innate immunity.

To confirm the importance of fat body–derived candidate regenerative proteins in disc regeneration, we conducted RNAi screening of 18 genes among 20 candidates (Fig. 3, *F* and *G*). Most of selected factors were required for disc regeneration. Fat body–specific RNAi knockdown of iron or immune-related factors, transferrin 1 (*tsf1*), *Fer1HCH*, *Fer2LCH*, peptidoglycan recognition protein SD (*PGRP-SD*), and necrotic (*nec*), had severe negative effects on disc regeneration (Fig. 3, *F* and *G*). These data indicate a nonautonomous requirement for innate immunity and iron-related factors for disc regeneration.

## Discussion

In this study, we found that fat body glycosylation was nonautonomously required for disc repair (Fig. 1*C*). To determine the humoral factors involved in disc repair, we established Mgat2-TbID, a Golgi-specific labeling tool. This system enabled us to perform secretome analysis involving the Golgi apparatus and identify secretory proteins crucial for disc regeneration, including innate immune regulators and iron mediators (Fig. 3, *D*–*F*). The contribution of immunity to tissue damage response and inflammatory regulation in tissue repair has been reported and discussed across species, such as mammals and flies (24, 25). In flies, hemocytes, one of the immune regulatory cells, are recruited to damaged wing discs and subsequently activate the JAK/STAT pathway in the hemocytes and fat body (26). However, it remains unclear how the Toll and Imd pathway-associated factors detected in the hemolymph proteomics play their respective roles. Recent *Drosophila* studies have suggested a link between iron and innate immunity. During septic injury, hemolymph iron is sequestered into the fat body, a phenomenon known as nutritional immunity (27). Iron uptake during infection in the fat body suppresses the growth of pathogenic bacteria. During this defense response, the iron-binding protein Tsf1 is secreted into the hemolymph to sequester serum iron, depending on the immune signaling of the Toll and Imd pathways. In our study, we found that innate immune factors, such as PGRP-SD, nec, and the iron-binding protein Tsf1, contributed to disc repair (Fig. 3*F*). Tsf1 is transcriptionally regulated by Toll and Imd signaling (27), nec is an inhibitor of Toll pathway (28), and PGRP-SD is an activator of Imd pathways (29, 30). These findings suggest that iron metabolism may be regulated by innate immune pathway. Further investigation is essential to elucidate the role and relationship between iron mediators and immune signaling in disc regeneration.

As previously mentioned, Tsf is an iron-binding protein, and tsf1 is one of the candidate proteins in this disc regeneration study. In insects, Tsf1 can bind ferric iron, and ferritin can store approximately 3000 ferric ions in the ferritin shell. They transport or uptake iron from various organs (31, 32). In mammals, iron uptake and release by blood cells play a role in tissue injury (33, 34). In injured cell populations, damage mitigation and repair responses *via* iron regulation by blood cells proceed locally to the injury site. In the early stages of injury, macrophages protect themselves from harmful iron released from the injured site and limit bacterial growth by absorbing and sequestering iron at the injury site. Such iron sequestration leads to anemia of inflammation observed in chronic inflammatory diseases, such as chronic infections, autoimmune diseases, cancer, and chronic kidney disease (33, 34). Subsequently, during the repair phase, macrophages release iron around the site of repair, stimulating cell proliferation and angiogenesis at the injury site (34). Thus, in mammalian tissue repair, the process begins with iron sequestration by macrophages, followed by the subsequent iron supply. Kupffer cells in the liver, like macrophages, are major Tsf producers and are central to systemic iron metabolism (34). However, it has not been shown how iron metabolism regulation by the liver affects remote tissue repair. In this study, we found that iron metabolism regulators generated through secretory pathway in the fat body remotely contribute to tissue repair (Fig. 3). How fat body–derived iron regulators affect hemocytes remains unclear and warrants further investigation.

Previous studies have examined the role of glycosylation in tissue regeneration, but glycosylation does not always promote regeneration. For instance, knock-out of two glycosyltransferases hinders axon regeneration in *Caenorhabditis elegans* (35). In contrast, in zebrafish hair cell regeneration, the repair process is enhanced by deficiency of Mgat5, an N-glycosylation enzyme (36). In mouse heart regeneration, a deficiency in N-glycosylation of hFSTL1 promotes regeneration (37). As shown above, glycosylation shows different requirements in tissue repair depending on factors, conditions, species, and tissues. In this study, we attempted to comprehensively search for the fat body–derived secretory proteins that influence disc regeneration using Mgat2-TbID (Fig. 3, *D* and *E*). Mgat2-TbID allowed us to identify humoral factors secreted *via* the Golgi apparatus. Glycosylation affects not only secretion but also protein structure, activity, and stability. In addition to a comprehensive search for secreted proteins, a deeper understanding of regenerative factors will require further investigation into glycosylation, including glycoproteomic approaches, such as pGlyco3 (38).

The fat body serves as a major endocrine organ, comparable to the liver and adipose tissue in mammals, and is responsible for most protein production through the secretory pathway (39). In this study, we employed a proximity-dependent secretory protein labeling method to investigate the secretory pathway proteins during disc regeneration. In previous studies, labeling enzymes were fused to the ER lumen to create proximity-dependent secretory protein labeling tools, such as ER-BioID (40), BirA∗G3-ER (2), and ER-TbID (41, 42, 43, 44). Using these tools, most secretory proteins from specific organs have been identified in the hemolymph or blood. In summary, we generated Mgat2-TbID as a chimeric form of a complex-type N-glycosylation enzyme as a tool to investigate the potential *in vivo* targets of Mgat2 or secreted factors passing through Golgi apparatus. We observed that Mgat2-TbID labeled Golgi proteins more efficiently than BirA∗G3-ER. Furthermore, a higher number of membrane proteins was detected in Mgat2-TbID than in BirA∗G3-ER (Table S1). We utilized V5-TbID as a control; however, V5-TbID was detectable in the hemolymph. Because V5-TbID lacks a signal peptide, the mechanism underlying its appearance in the hemolymph remains unclear. Importantly, proteomic analyses showed that ER- and Golgi-targeted TbID preferentially labeled secreted proteins compared to V5-TbID, suggesting that cytosolic TbID does not efficiently access the secretory pathway. Thus, while we acknowledge this as a limitation, we do not expect hemolymph V5-TbID to substantially confound our proteomic conclusions. Considering these observations, our Golgi-specific labeling platform enhances the specificity of proximity labeling within the secretory pathway and is widely applicable for future studies on inter-organ protein trafficking and membrane protein labeling.

## Experimental procedures

### Fly strains and genetics

All flies were reared on Saf-instant and oriental corn meal medium (hereafter Saf or oriental food) at 25 °C. The detailed food composition per liter of Saf and Oriental foods is shown here. Per liter, the Saf-food contained agar (8 g; KISHIDA CHEMICAL, #260-01705), glucose (60 g; WAKO, #049-31165), dry yeast (60 g; Saf-instant RED (Saf-instant baker’s yeast)), corn flour (40 g; NIPPN, #100016), propionic acid (3 ml; WAKO, #163-04726), and 10% butyl p-hydroxybenzoate (1.5 ml; WAKO, #132-02635). The Oriental food contained agar (8 g; KISHIDA CHEMICAL, #260-01705), glucose (100 g; WAKO, #049-31165), dry yeast (40 g; Oriental), corn flour (40 g; NIPPN, #100016), propionic acid (3 ml; WAKO, #163-04726), and 10% butyl p-hydroxybenzoate (5 ml; WAKO, #132-02635) per liter. The fly lines and genotypes used in this study are presented in Tables S5 and S6.

### Temperature shift protocol for temporal ablation

Embryos were laid at 25 °C for 4 h, and we defined this time point as after egg laying (AEL) as 0 h. The temperature was maintained at 25 °C and shifted to 29 °C, 24 h after AEL 0 h. For ablation with *WP*^*QF2*^ *> DtA*^*ts*^, the temperature was raised to 29 °C for 54 h, and shifted to 18 °C from the early third instar larval stage. After maintaining an 18 °C for 38 h, the temperature was returned to 29 °C and flies were either allowed to pupate and eclose, or were dissected at the time points indicated as 0, 6, or 30 h AA. For proteomics analyses, developmental speed measurements, and quantitative PCR analysis, larvae without DtA^ts^ were used as the nondamaged control, and samples were collected at 0 or 6 h NA, at the same time points as AA.

### Wing size assessment

The flies were preserved in a mixture of ethanol/glycerin (3:1). We observed wing phenotypes by microscopy and manually classified the wing size into four categories: 1) normal: intact wings; 2) chipped: chipped or crumpled wings having a size >90% of control; 3) mild: wings with intermediate phenotypes, or between “chipped” and “severe” groups, 4) severe: wings measuring <30% of normal size. When counting the wing regeneration rate, we noted the statistical analysis values and number of samples (N) above each genotype bar. To take wing pictures, we dehydrated flies in 100% ethanol for 4 to 24 h, following dissection of wings in 100% ethanol and mounting them in Euparal mounting medium (WALDECK GmbH & Co KG, DIVISION CHROMA, 35358-86). Images were captured with a Leica microscope DM5000.

### Quantification of developmental speed

Time to pupation was measured to determine the developmental speed. The injured and uninjured larvae were subjected to temperature changes according to temperature shift protocol, and the number of pupated flies was subsequently counted at 10 time points of AEL at 96, 105, 120, 129, 144, 153, 168, 177, 192, and 201 h. Pupae number at AEL of 201 h was set to 1.0, and the developmental speed from larvae to pupae was measured as the pupal fraction at each time point.

### Immunohistochemistry

Discs, whole larvae, fat body, and salivary glands were dissected in PBS and fixed with a 4% paraformaldehyde PBS solution for 20 min. Discs were washed with 0.1% Triton X-100 PBS solution (PBST). Fat body, whole larvae, and salivary glands were washed with 0.3% Triton X-100 PBST. The antibodies used were as follows: rabbit anti-cleaved Dcp1 (Asp216) 1:200 (Cell Signaling, 9578S), mouse anti-Wg (4D4) 1:500 (Developmental Studies Hybridoma Bank), rat anti-phospho-histone H3 (Phospho S28) 1:500 (Abcam, ab10543), rabbit anti-RFP 1:500 (Abcam, ab62341), and mouse anti-V5 1:500 (Invitrogen, R96025). The secondary antibodies used in this study were as follows: Alexa Fluor antibodies (Thermo Fisher Scientific, A-21202, A-21206, A-21208, A-31571, A-31573, A-48272) and AffiniPure antibodies (Jackson ImmunoResearch, 715-165-150, 711-165-152, 712-166-153) diluted at 1:500. The probes used were as follows: Hoechst 33342 (H3570) and Rhodamine Phalloidin (R415) diluted 1:200 (Life Technologies) and Streptavidin-Cy3 (Jackson ImmunoResearch, 016-160-084) diluted 1:500. Confocal images were captured using a Leica SP8 microscope. Quantification of pH3-positive cells and cell death in the WP region, and RedStinger intensity in the wing discs, was performed using Fiji software. WP region is the area of the WP labeled with WP^QF2^ and QUAS-mCD8-GFP in the wing disc. pH3-positive cells were normalized to WP size, as indicated by *WP*^*QF2*^*>mCD8-GFP* cells, and RedStinger intensity was normalized to wing disc size, as indicated by Hoechst 33342 staining. Pearson’s correlation coefficients were calculated using the Coloc 2 plugin in Fiji software (ImageJ2, version 2.16.0/1.54p, https://fiji.sc/). Both the whole-image (“no-threshold”) Pearson’s R and the thresholded (“above-threshold”) Pearson’s R were reported. For the thresholded analysis, thresholds were determined automatically using the Costes method implemented in Coloc 2.

### Fly RNA extraction and complementary DNA synthesis

RNA extraction was performed using the RNeasy Micro Kit (QIAGEN) and the ReliaPrep RNA Tissue Miniprep System (Promega) according to the manufacturer’s instructions. Rverse transcription quantitative PCR was performed according to the protocol for nonfibrous tissues using the ReliaPrep RNA Tissue Miniprep System (Promega). Briefly, the fat body of three third-instar larvae were dissected in PBS and homogenized in 1.5 ml tubes containing lysis buffer. Then, the samples were stored at −80 °C according to the protocol of ReliaPrep RNA Tissue Miniprep System (Promega). Complementary DNA (cDNA) was synthesized from 400 ng of total RNA using PrimeScript RT Master Mix (Perfect Real Time) (Takara Bio).

### Expression constructs

cDNA samples for construction were prepared according to the RNeasy Micro Kit protocol (QIAGEN, 74004) and PrimeScript RT Master Mix (Perfect Real Time) (Takara Bio, RR036A). In QIAGEN protocol, six adults or six *UAS-lacZ*^*RNAi*^ larvae were homogenized in 150 μl of QIAzol, followed by the addition of 350 μl QIAzol and incubation for 30 min. Subsequently, samples were stored at −80 °C according to the protocol of RNeasy Micro Kit (QIAGEN, 74004). For constructing *UAS-Mgat2-V5-TurboID*, the isoform RB of *Mgat2* was cloned from the cDNA extracted from adult or third instar larvae of the *UAS-lacZ*^*RNAi*^ using forward primer 5′- AAATCAAAGGATCCCCAAAATGTCGAAAATG and the reverse primer 5′- GGGGATGGGCTTGCCCCTCGTGGCCAGCGTC *UAS-Mgat2-V5-TurboID* and *UAS-V5-TurboID* were constructed using the *pAct5-V5-TurboID* plasmid, which was generously provided by Dr Shinoda of the University of Tokyo (45). The V5-TurboID fragment for UAS-V5-TurboID was amplified from the *pAct5-V5-TurboID* plasmid, by PCR using the forward primer 5′- AAATCAAAGGATCCCCAAAATGGGCAAGCCC and the reverse primer 5′- TAGTGGTACCCTCGATCACTGCAGCTTTTC.

The V5-TurboID fragment for UAS-Mgat2-V5-TurboID was amplified from the *pAct5-V5-TurboID* plasmid, by PCR using the forward primer 5′- GGCAAGCCCATCCCCAACCCC.

The reverse primer had the same sequence as that of The V5-TurboID fragment for *UAS-V5-TurboID*.

The amplified fragments were inserted into *pUASz1.0* (DGRC, #1431) vector with *Xho*I restriction using the NEBuilder HiFi DNA Assembly Master Mix. This sequence was confirmed by Eurofins, Inc. The vector was injected into the transgenic *y1 w67c23; P{CaryP}attP40* strain. Transgenic flies were generated by Best Gene Inc.

### Quantitative RT-PCR

cDNA was diluted tenfold with Milli-Q, and rverse transcription quantitative PCR was performed using TB Green Premix Ex Taq II (Tli RNaseH Plus) (Takara Bio, RR820L) to a total volume of 10 μl. Quantitative PCR was performed using the Quantstudio 6 Flex Real-Time PCR system (Thermo Fisher Scientific) according to the standard protocol provided with the software. The primer sequences are as follows:

RNA pol II Forward primer 5′- CCTTCAGGAGTACGGCTATCATCT.

RNA pol II Reverse primer 5′- CCAGGAAGACCTGAGCATTAATCT.

Mgat2 Forward primer 5′- ATGTCGAAAATGAGGGGTCGC.

Mgat2 Reverse primer 5′- ACTCCGCATGTAGAAGTTGTTG.

Fused Lobes Forward primer 5′- TTCATCCTGACGGTGCTCTAC.

Fused Lobes Reverse primer 5′- GTATGGGAAAGCCACTAGCATC.

Mgat4b Forward primer 5′- CAGTTCGTGATTGGAGTACCC.

Mgat4b Reverse primer 5′- GGATCAGACAGTCCACGGTT.

Mgat3 Forward primer 5′- ATGCAGATGTGAAAGTGGCTG.

Mgat3 Reverse primer 5′- GTGCTACCGCCTCGTTTACTG.

Mgat1 Forward primer 5′- CCAAGAACGTGTTTGAGTTCGT.

Mgat1 Reverse primer 5′- CAGCTCCGCCGATATTTCG.

### Western blotting

Fat body and hemolymph from 10 to 20 larvae were collected. Fat body was collected in 55 μl ofradioimmunoprecipitation assay (RIPA) buffer (1 M Tris–HCl, pH 8.0 50 ml/5 M NaCl 30 ml/5 g sodium deoxycholate/1 g SDS/NP-40 10 ml/MQ up to 1 L) containing 1x protease inhibitor (cOmplete, EDTA-free Protease Inhibitor Cocktail [Roche, 16829900]). After centrifugation at 10,000×*g*, 4 °C for 10 min, 45 μl of the intermediate layer was collected and stored at −80 °C. Larval hemolymph was collected in 15 μl ice-cold PBS by pinching the epidermis using forceps and then the extracts were mixed with 30 μl of PBS with 1x protease inhibitor (cOmplete, EDTA-free Protease Inhibitor Cocktail [Roche, 16829900]). After centrifugation at 1000×*g*, 4 °C for 3 min to remove hemocytes, 40 μl of the supernatant was collected and stored at −80 °C. After protein quantification using the PierceBicinchoninic Acid Protein Assay Kit (Thermo Fisher Scientific, 23225), 6 × Laemmli sample buffer (1 M Tris–HCl pH 6.8, 12% SDS, 0.6% bromophenol blue, 15% 2-ME) was added, and samples were boiled at 95 °C for 5 min and were adjusted to the same concentration. An equal amount of total protein was separated by 10 or 12.5% SDS-PAGE. The proteins were then transferred to Immobilon-P PVDF membranes (Millipore, IPVH00010) at 25 V, 2.5 mA for 20 min using the Trans-Blot Turbo Transfer System (Bio-Rad, 1704150). Membranes for total protein visualization were stained with Ponceau-S (Funakoshi, BCL-PSS-01) according to the manufacturer’s instructions. The membranes for streptavidin blotting were blocked with 3% BSA in Tris-buffered saline with Tween 20 (TBST) for 10 min. Then, streptavidin-horseradish peroxidase (HRP) (Thermo Fisher Scientific, 19-534-050) was diluted at 1:20,000 in 3% BSA and treated on the membrane for 1 to 2 h at room temperature. The membranes for antibody staining were blocked with 4% Difco skim milk (Becton, Dickinson and Company, 232100)/TBST for 10 min at room temperature. Primary antibodies were diluted in 4% Difco skim milk and treated on the membrane overnight at 4 °C. The primary antibodies used were as follows: rabbit anti-Egr 1:1000 (generated in (46)), mouse anti-V5 1:5000 (Invitrogen, R96025), and mouse alpha-tubulin 1:5000 (Cell Signaling, 3873S). After three washes with TBST at room temperature, the HRP-conjugated secondary antibody was diluted in 4% skim milk and treated on the membrane for 1 to 2 h at room temperature. The HRP-conjugated secondary antibodies used in this study were as follows: anti-rabbit IgG, HRP-linked antibody (Cell Signaling, 7074S), and anti-mouse IgG (H + L) HRP conjugate (Promega, W402B) diluted 1:10,000. The HRP-conjugated secondary antibody was removed, washed three times with TBST at room temperature, and then treated with Immobilon Western Chemiluminescent HRP Substrate (Millipore, WBKLS0500). Signals were detected using FUSION SOLO 7S. EDGE (Vilber-Lourmat). To quantify the Egr and α-tubulin immunoblot signals in Fig. S2*C*′, bands at ∼35 kDa (Egr) and ∼51 kDa (α-tubulin) were selected as regions of interest and quantified by the Gel Analysis tool in Fiji. Background signal was subtracted using an adjacent area of the same lane. Egr band intensities were then normalized to the corresponding α-tubulin intensities.

### PNGase F treatment

The PNGase F PRIME treatment was performed according to the manufacturer’s instructions (Funakoshi, NZS1). Briefly, 25 μg of the proteins extracted from fat body described in “Western Blotting” was diluted in 1x PBS to a final volume of 11 μl. Then, 1 μl of 5% SDS (FUJIFILM Wako Pure Chemical Corporation, 191-07145) and 1 μl of 1 M DTT (FUJIFILM Wako Pure Chemical Corporation, 045-08974) were added. Samples were denatured by heating to 95 °C for 10 min and then cooled on ice. Next, 2 μl of 10% NP-40 (FUJIFILM Wako Pure Chemical Corporation, 142-10061) and 1 μl of recombinant PNGaseF PRIME or MilliQ (as PNGase F negative control) were added to the samples, followed by incubation at 37 °C for 30 min.

### *Ex vivo* TurboID incubation

Schneider’s *Drosophila* Medium (Thermo Fisher Scientific, 21720024) was supplemented with 50 μM biotin (FUJIFILM Wako Pure Chemical Corporation, 029-08713). For ATP (+) samples, 100 μM ATP (FUJIFILM Wako Pure Chemical Corporation, SS-9211) was added; for ATP (−) controls, an equal volume of Milli-Q water was added. The medium was further supplemented with 1× protease inhibitor cocktail (cOmplete, EDTA-free Protease Inhibitor Cocktail; Roche, 16829900) and kept ice-cold until use. Hemolymph was collected from 10 larvae. Larval hemolymph was extracted into 15 μl of the ice-cold supplemented medium by gently pinching the epidermis with forceps, and the extract was then mixed with an additional 30 μl of the same medium. After centrifugation at 1000×*g* for 3 min at 4 °C to remove hemocytes, 40 μl of the supernatant was collected. The hemolymph samples were incubated at 25 °C for 1 h and subsequently stored at −80 °C.

### Purification of biotinylated proteins

Biotin food was prepared by adding 1 mM Biotin (FUJIFILM Wako Pure Chemical Corporation, 132-02635) to saf food to a final concentration of 100 μM. Animals were placed in vials containing biotin-containing food for injury experiments. Samples were extracted as described in the Western blotting section. Purification of the labeled proteins was conducted as previously described (45) with some modifications. Briefly, 150 μg of biotinylated protein-containing lysate was subjected to FG-NeutrAvidin beads (Tamagawa Seiki Co., Ltd, TAS8848 N1171) purification. FG-NeutrAvidin beads (25 μl, approximately 500 μg) were washed three times with RIPA buffer. Benzonase (Merck, 70746-4)-treated biotinylated protein samples suspended in 1 ml RIPA buffer were incubated overnight at 4 °C. Conjugated beads were magnetically isolated and washed with 500 μl of ice-cold RIPA buffer solution, 1 M KCl solution, 0.1 M Na_2_CO_3_ solution, and 4 M urea solution. For LC–tandem mass spectrometry (MS/MS) analysis, the purified samples were washed with 500 μl ultrapure water (FUJIFILM Wako Pure Chemical Corporation, 214-01301) and 500 μl of 50 mM ammonium bicarbonate (Sigma-Aldrich, A6141-25G). The samples were then mixed with 50 μl of 0.1% RapiGest SF (Waters, 186001861) as anionic surfactant diluted in 50 mM ammonium bicarbonate, and 5 μl of 50 mM Tris(2-carboxyethyl) phosphine hydrochloride (Sigma, C4706) was subsequently added as a reducing agent. The samples were incubated at 60 °C for 5 min, and then 2.5 μl of 200 mM methyl methanethiosulfonate (Thermo Fisher Scientific, 23011) was added. One microgram sequence-grade modified trypsin (Promega, V5111) was then added for on-bead trypsin digestion at 37 °C for 16 h. The beads were then magnetically isolated and 60 μl of the supernatants was collected. Then, 3 μl of 10% TFA (FUJIFILM Wako Pure Chemical Corporation, 206-10731) was added to the supernatants, and the samples were incubated at 37 °C for 60 min with gentle agitation. The samples were then centrifuged at 20,000×*g*, 4 °C for 10 min. The peptides were desalted and purified using a GL-tip SDB (GL Sciences, 7820-11200) following the manufacturer's instructions. The samples were vacuum dried at 45 °C for 30 min (Tommy Seiko, CC-105) and dissolved in 25 μl of 0.1% formic acid (Kanto Chemical, 16245-63). The samples were then centrifuged at 20,000×*g*, 4 °C for 10 min, and the supernatants were collected. The peptide concentrations were determined using the bicinchoninic acid assay (Thermo Fisher Scientific, 23225). Finally, 250 ng of the purified protein was subjected to LC–MS/MS analysis.

### LC-MS/MS analysis

Samples were loaded onto an Acclaim PepMap 100 C18 column (75 μm × 2 cm, 3 μm particle size and 100 Å pore size; Thermo Fisher Scientific, 164946) and separated using a nano-capillary C18 column (75 μm × 12.5 cm, 3 μm particle size, Nikkyo Technos, NTCC-360/75-3-125) in an EASY-nLC 1200 system (Thermo Fisher Scientific). The elution conditions are listed in Table S7. The separated peptides were analyzed using QExactive (Thermo Fisher Scientific) in the data-dependent MS/MS mode. The parameters for MS/MS analysis are listed in Table S7. The collected data were analyzed using Proteome Discoverer (PD) 2.2 software with the Sequest HT search engine (https://knowledge1.thermofisher.com/Software_and_Downloads/Chromatography_and_Mass_Spectrometry_Software/Proteome_Discoverer/Proteome_Discoverer_User_Guides/Proteome_Discoverer_2.2_overview). The parameters for the PD 2.2 analysis are listed in Tables S7. Peptides were filtered at a false discovery rate of 0.01 using the Percolator node. Label-free quantification was performed based on the intensities of the precursor ions using a precursor-ion quantifier node. Normalization was performed using the total amount of peptides in all average scaling modes. Proteins with 1.5 or 2-fold higher abundance ratios relative to the lacZ or V5-TbID controls were considered for further analysis. The mass spectrometry proteomics data were deposited in the ProteomeXchange Consortium *via* the jPOST partner repository with the dataset identifier PXD054313 for fat body samples and PXD055017 for hemolymph samples.

### Analysis of secreted regenerative proteins based on proteomics data

For the analysis of proteomics abundance ratio, proteins with Mgat2-TbID/V5-TbID were visualized by a volcano plot using the R software, and those proteins with a value higher than 2.0 and a *p* value below 0.05 were selected. Candidate proteins detected in the injury condition were extracted. Functional annotation, visualization, and integrated discovery were performed using the web tool DAVID (https://david.ncifcrf. gov/). 3D correlation plots were visualized using the R software (version 4.3.1, https://cran.r-project.org/src/base/R-4/).

### Statistical analysis

Data are presented as stacked bar graphs, bar charts, or mean ± SD. To analyze the independence of the cross-tabulation tables used in the stacked bar graphs, *p* values were calculated using Fisher's exact test using the R software for all data. Fisher’s exact test was applied to the 2 × 4 table and to define the four categories used for the contingency analysis. Each analysis was compared with the control of *lacZ*^*RNAi*^. The unpaired *t* test was used to calculate *p* values for comparisons between two groups, while one-way ANOVA with multiple comparisons was used to calculate *p* values for analyses involving more than two groups. *p* values are NS: not significant, ∗: *p* < 0.05, ∗∗: *p* < 0.01, ∗∗∗: *p* < 0.001, and ∗∗∗∗: *p* < 0.0001. GraphPad Prism statistical analysis software (version 8, https://www.graphpad.com/updates/prism-8-release-notes) and RStudio software (version 2023.06.1+524, https://dailies.rstudio.com/version/2023.06.1%2B524/) were used to generate graphs and perform statistical analyses.

## Data availability

The representative data produced for this work are contained within the article. The mass spectrometry proteomics data for this work were deposited in the ProteomeXchange Consortium *via* the jPOST partner repository with the dataset identifier PXD054313 for fat body samples and PXD055017 for hemolymph samples.

## Supporting information

This article contains supporting information (47, 48, 49).

## Conflict of interest

The authors declare that they have no conflicts of interest with the contents of this article.
