# Supplementary material for: Proximal labeling of the Golgi secretome reveals fat body–derived humoral factors in Drosophila disc regeneration
Source: J Biol Chem. 2026 May 27;302(7):113198. doi: 10.1016/j.jbc.2026.113198 (PMC13310645; doi:10.1016/j.jbc.2026.113198)
Supplement: Supporting Figures [file mmc8.docx]

**Supporting Information**

**Proximal Labeling of the Golgi Secretome Reveals Fat Body-Derived Humoral Factors in *Drosophila* Disc Regeneration**

Yutaka Yoshida1, Soshiro Kashio1,2*, and Masayuki Miura1,3,4*

1Department of Genetics, Graduate School of Pharmaceutical Sciences, The University of Tokyo, 7-3-1 Hongo, Bunkyo-ku, Tokyo 113-0033, Japan; 2 Ubiquitin Biology Laboratory, Graduate School of Frontier Biosciences, The University of Osaka, Yamadaoka 1-3, Suita, Osaka 565-0871, Japan; 3Laboratory for Cell Vigor Regulation, National Institute for Basic Biology, Nishigonaka 38, Okazaki, Aichi 444-8585, Japan; ^4^Basic Biology Program, The Graduate University for Advanced Studies, SOKENDAI, Nishigonaka 38, Okazaki, Aichi 444-8585, Japan

*Corresponding authors:

Soshiro Kashio

**Email:** kashio.soshiro.fbs@osaka-u.ac.jp

Masayuki Miura

**Email:** miura@nibb.ac.jp

**Figure S1**


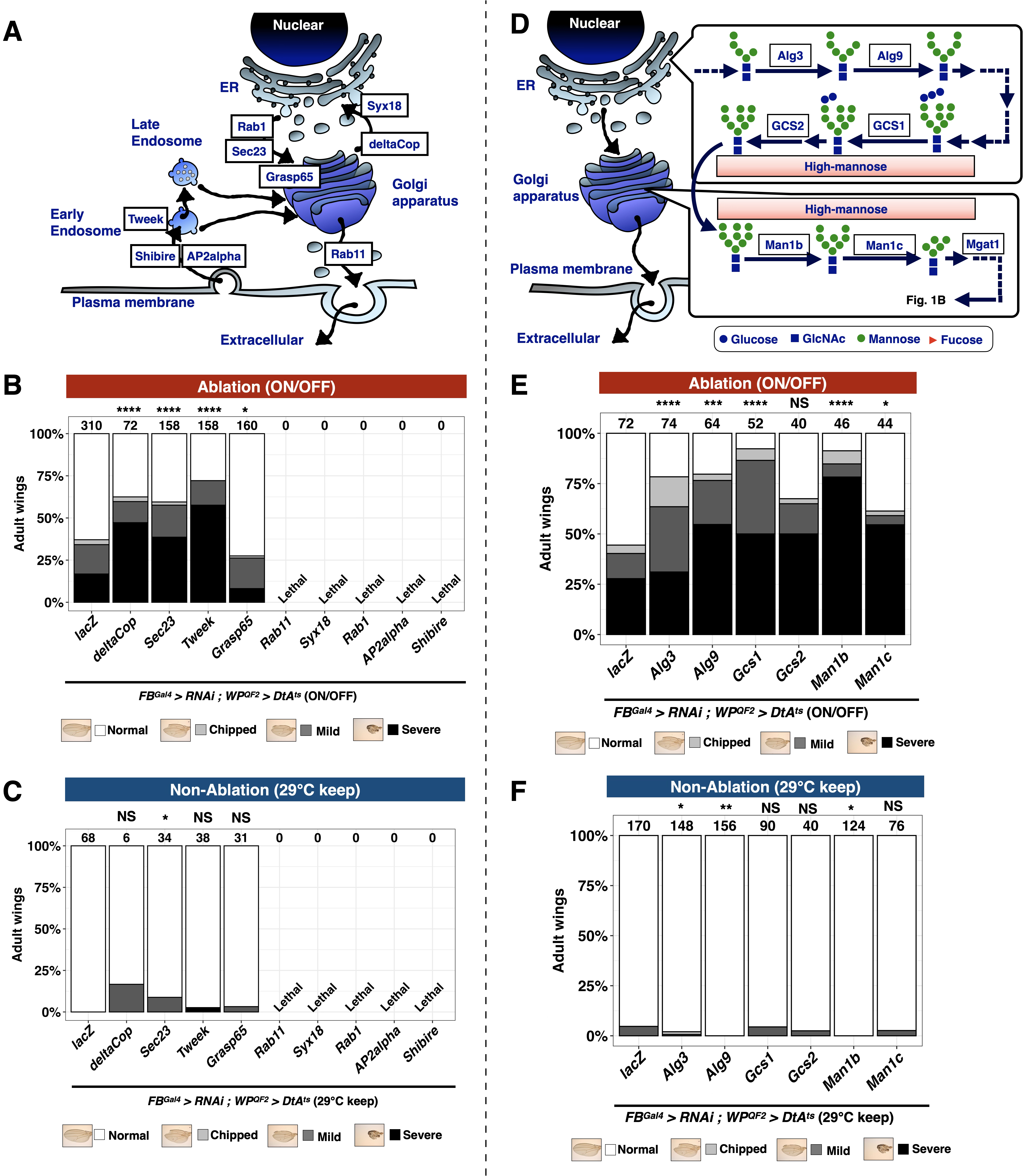


**Figure S1　Requirement of general secretory components in fat body for disc regeneration**

(A) Protein trafficking pathway, including secretory pathway and endocytosis.

(B and C) Comparison of adult wing sizes between ablation (B) and non-ablation (C). RNAi knockdown of protein trafficking components hampered disc regeneration. RNAi knockdown was induced in fat body by *FB^Gal4^*. Statistical analysis was conducted using Fisher’s exact test to compare control (*WP^QF2^>DtA^ts^; FB^Gal4^>lacZ^RNAi^*) with treated larvae. NS: not significant, *: p<0.05, ****: p<0.0001. Number of flies is listed above each genotype in the bar graph.

(D) Pathway of N-glycosylation enzymes, including ER. Detailed sequential steps after Mgat1 in the Golgi apparatus were shown in Fig. 1B.

(E and F) Comparison of adult wing sizes between ablation (E) and non-ablation (F). RNAi knockdown of N-glycosylation enzymes hampered disc regeneration. RNAi knockdown was induced in fat body by *FB^Gal4^*. Statistical analysis was conducted using Fisher’s exact test to compare control (*WP^QF2^>DtA^ts^; FB^Gal4^>lacZ^RNAi^*) with treated larvae. NS: not significant, *: p<0.05, **: p<0.01, ***: p<0.001, ****: p<0.0001. Number of flies is listed above each genotype in the bar graph.

**Figure S2**


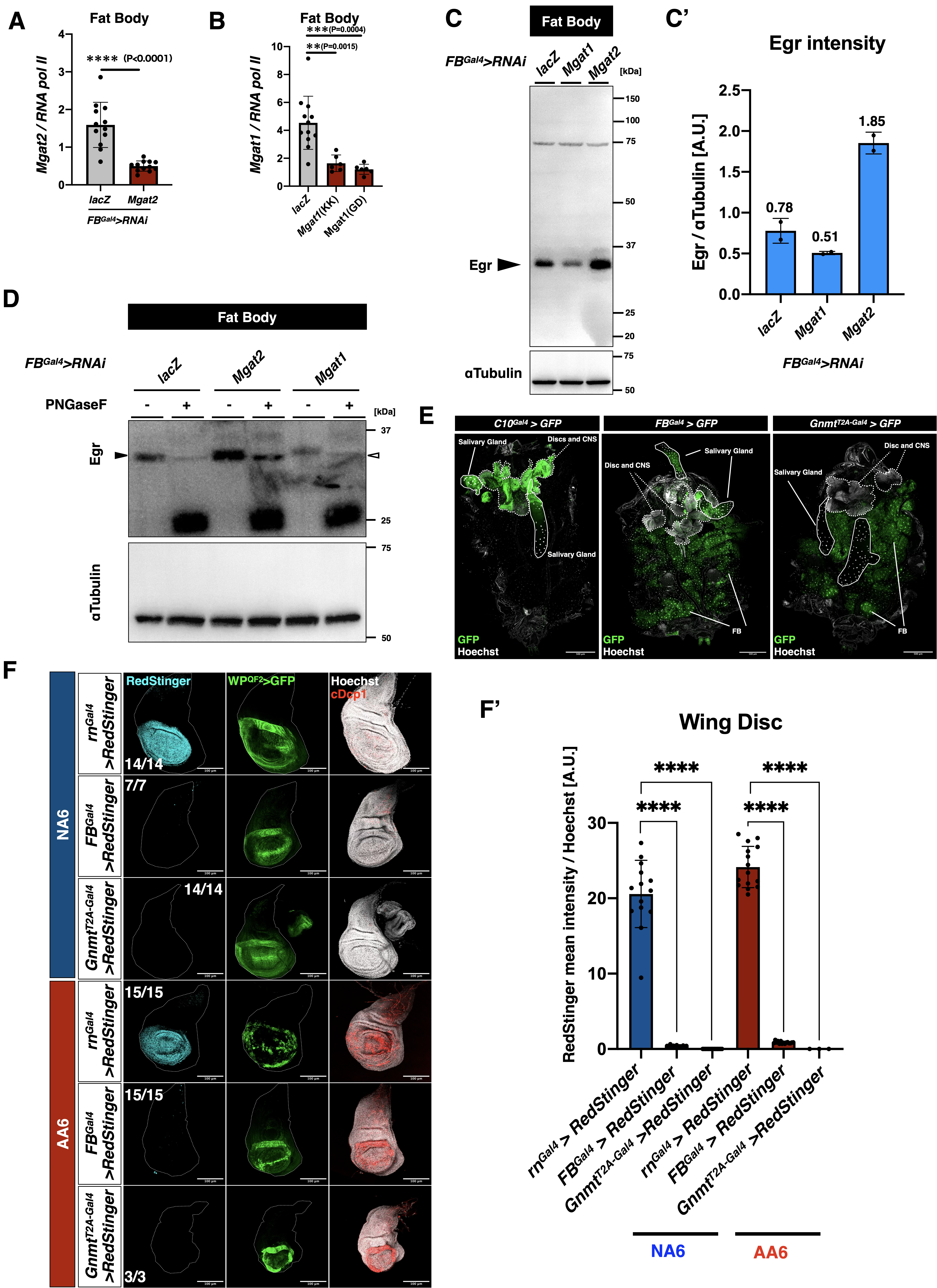


**Figure S2** **Effects of *Mgat1* and *Mgat2* RNAi knockdown and fat body-specific Gal4 expression**

(A) Expression level of *Mgat2* in non-ablated larval fat body. RNAi knockdown of *Mgat2* in fat body using *FB^Gal4^*. mRNA expression was normalized with *RNA pol II* expression. An unpaired t-test was applied. ****: p<0.0001. Number of biological replicates is n=12.

(B) *Mgat1* expression level in the non-ablated larval fat body. RNAi knockdown of *Mgat1* in fat body using *FB^Gal4^*. Each mRNA expression was normalized with *RNA pol II* expression. An unpaired t-test was applied. **: p<0.01, and ***: p<0.001. Number of samples was n=12 in *lacZ^RNAi^* and n=6 in *Mgat1^RNAi^*.

(C. C’) Western Blotting of Eiger (Egr) proteins under fat body specific *Mgat1* or *Mgat2* RNAi knockdown conditions. αTubulin was used as the internal control. The black arrowhead indicates the secreted form of Egr. (C) indicates the data of WB, and (C’) is the quantification of (C) by normalizing the Egr band from αTubulin. Mean intensity of each genotype is: *lacZ^RNAi^* = 0.78, *Mgat1^RNAi^* = 0.51, and *Mgat2^RNAi^* = 1.85. The number of biological replicates was n = 2 for all conditions.

(D) Western blot of the Egr proteins under fat body-specific *Mgat1* or *Mgat2* RNAi knockdown conditions and in the presence or absence of PNGase F treatment. αTubulin was used as the internal control. The black arrowhead indicates the secreted form of Egr, and the white arrowhead indicates the PNGase F-treated (deglycosylated) form. Number of biological replicates was n = 2 in all conditions.

(E) Gal4 expression patterns were examined by using *UAS-GFP* with fat body-specific Gal4, *FB^Gal4^*, and *Gnmt^T2A-Gal4^* (47). *C10^Gal4^* was used as a control driver for wing disc, salivary gland, and neuron. Gal4 expression in whole larvae was shown. GFP was indicated as green, and Hoechst was indicated as white. Number of samples was n=2 in *FB^Gal4^* and *Gnmt^T2A-Gal4^*, and n=1 in *C10^Gal4^*.

(F, F’) Gal4 expression pattern in the regenerating wing disc was examined using *UAS-RedStinger* with fat body-specific Gal4, *FB^Gal4^*, and *Gnmt^T2A-Gal4^*. *rn^Gal4^* was used as a control for the wing pouch driver. Gal4 expression images in the wing discs are shown in (F), and quantification of (F) is shown in (F’). RedStinger is indicated as cyan, wing pouch region is indicated as green, dead cells (cleaved Dcp1 positive cells) are indicated as red, and Hoechst staining is indicated as white. Number of samples was n = 14 for *rn^Gal4^*, NA6 and *Gnmt^T2A-Gal4^*, NA6; n = 7 for *FB^Gal4^*, NA6; n = 15 for *rn^Gal4^*, AA6 and *FB^Gal4^*, AA6; and n = 3 for *Gnmt^T2A-Gal4^*, AA6.

**Figure S3**


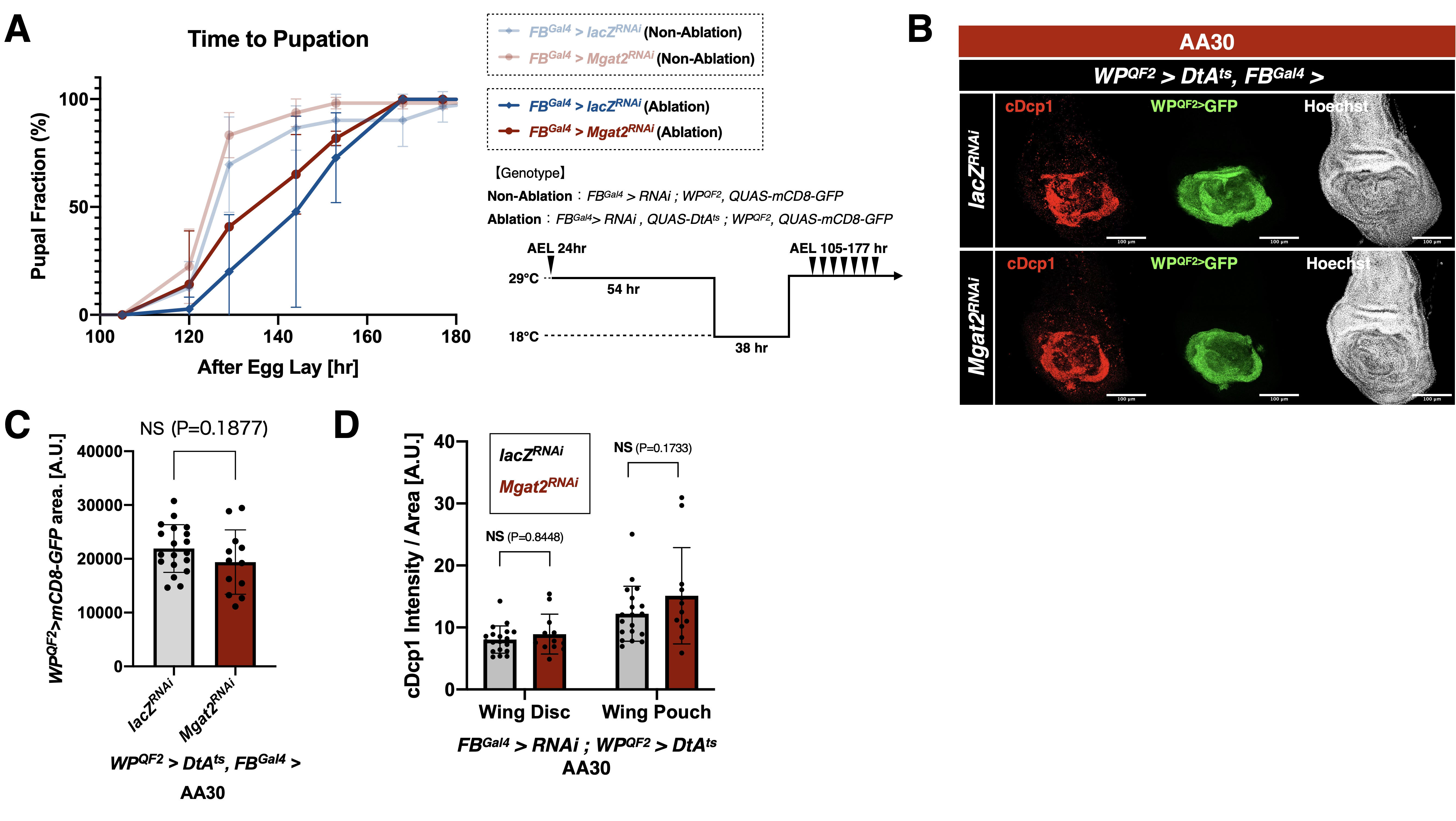


**Figure S3** 　**Mgat2 in fat body did not affect developmental delay and cell death caused by tissue injury in wing discs**

(A) Pupal fraction for *FB^Gal4^ > Mgat2^RNAi^* flies in ablation and non-ablation. n = 4 (Non-Ablation of *lacZ^RNAi^*), 5 (Non-Ablation of *Mgat2^RNAi^*), 4 (Ablation of *lacZ^RNAi^*), and 3 (Ablation of *Mgat2^RNAi^*), respectively, from top to bottom. Temperature treatment was similar between ablation and non-ablation.

(B) *WP^QF2^ > DtA^ts^* induced anti-cleaved Dcp1 (cDcp1) signal in ablated wing pouch region at AA30. White scale bar, 100 μm. Number of wing discs was n=19 in *lacZ^RNAi^* and n=12 in *Mgat2^RNAi^*.

(C) *WP^QF2^>mCD8-GFP* area of *FB^Gal4^ > lacZ^RNAi^ and Mgat2^RNAi^* at AA30 in (B) was quantified. Error bars indicate standard error of the mean. An unpaired t-test was applied. NS: not significant. Number of wing discs was n=19 in *lacZ^RNAi^* and n=12 in *Mgat2^RNAi^*.

(D) cDcp1 intensity in the wing pouch region and whole wing disc region in (B) was quantified. cDcp1-signal amount was unchanged between *FB^Gal4^ > lacZ^RNAi^* and *FB^Gal4^ > Mgat2^RNAi^*. Error bars indicate standard error of the mean. An unpaired t-test was applied. NS: not significant. Number of wing discs was n=19 in *lacZ^RNAi^* and n=12 in *Mgat2^RNAi^*.

**Figure S4**


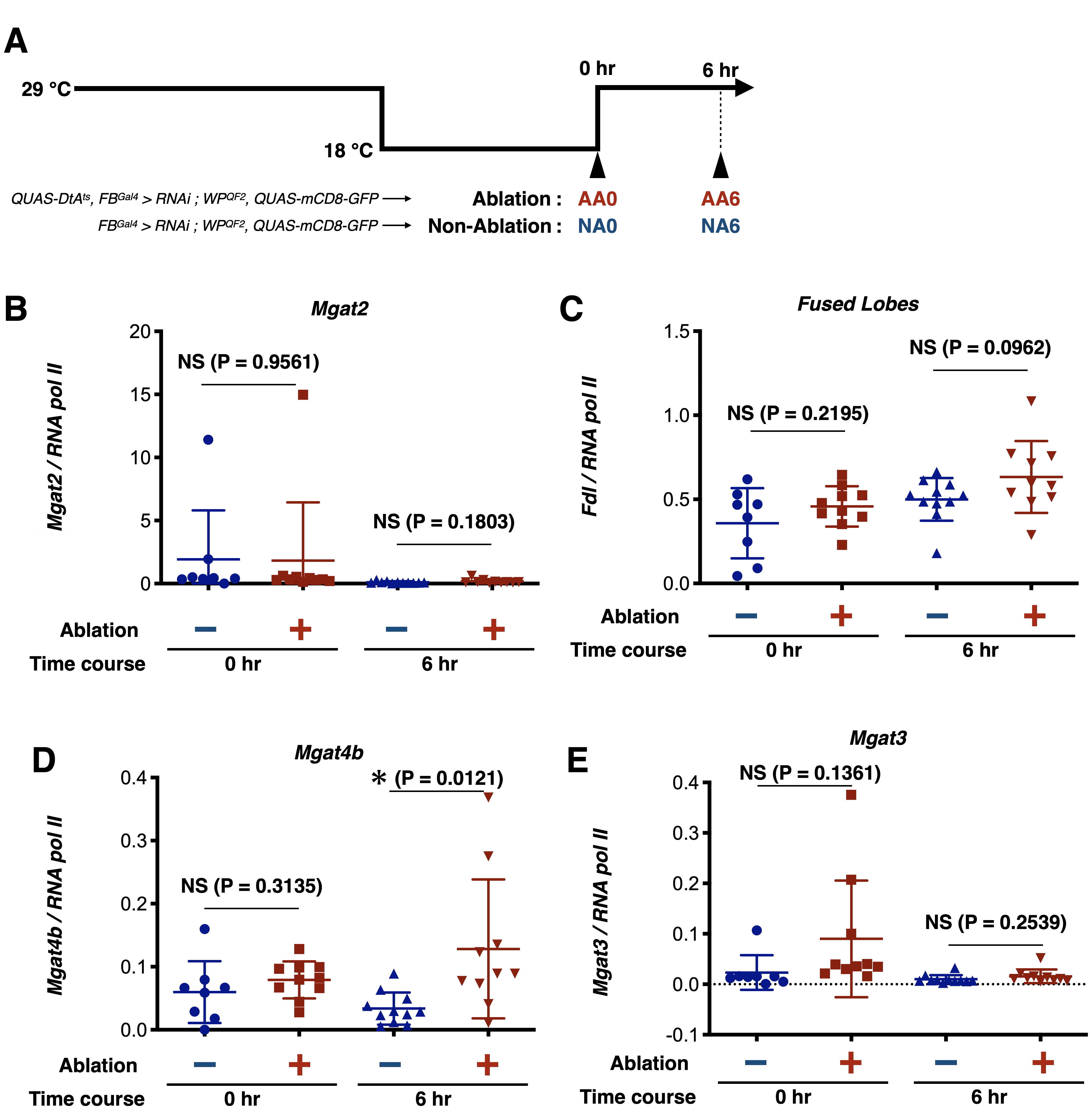


**Figure S4** 　**Expression of glycosyltransferases in fat body during disc regeneration**

(A) Scheme of the sampling time course. Sampling was performed at two time points: 0 and 6 h after ablation (AA). Ablation or Non-Ablation (NA) conditions were applied to DtA^ts^-expressing or non-DtA^ts^-expressing larvae, respectively, and they were maintained under the same temperature shift.

(B-E) *Mgat2*, *Fused Lobes*, *Mgat4b,* and *Mgat3* expression level in the disc-ablated and non-ablated larval fat body. Each mRNA expression was normalized with *RNA pol II* expression. One-way ANOVA and Tukey’s multiple comparison test were applied. NS: not significant, *: p<0.05. Number of samples was n=8 in NA0, n=10 in AA0, n=11 in NA6, and n=10 in AA6.

**Figure S5**


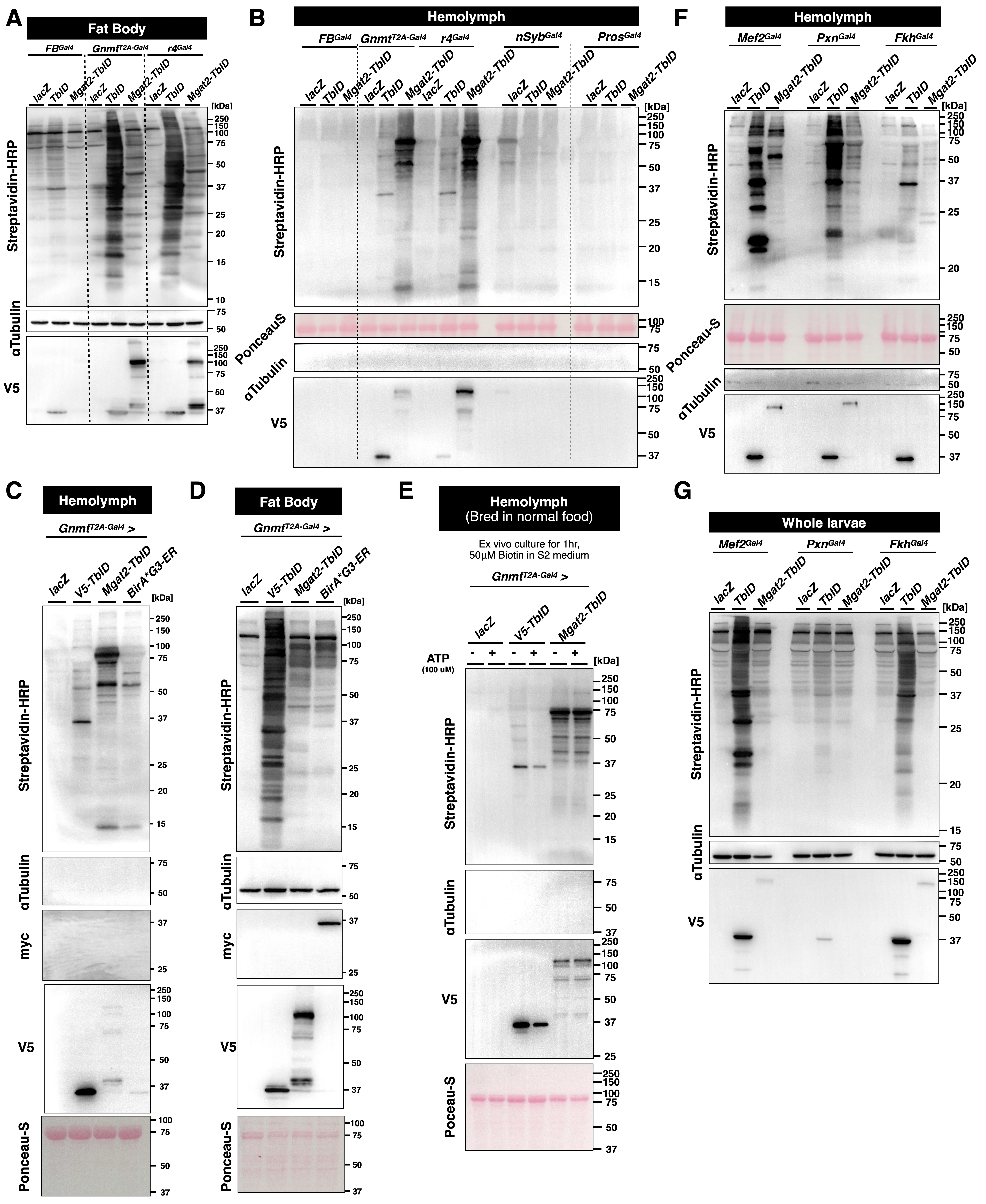


**Figure S5** **Validation of secretory protein labeling with Mgat2-TbID**

(A and B) Western blotting for biotinylated proteins (detected by Streptavidin-HRP) in fat body (A) and hemolymph (B). *LacZ*, *V5-TbID,* and *Mgat2-TbID* were expressed in fat body with *FB^Gal4^*, *Gnmt^T2A-Gal4^*, *r4^Gal4^*, in neurons with *nSyb^Gal4^*, and neural cells and gut enteroendocrine cells with *Pros^Gal4^*. Samples were collected in Non-Ablation (NA) condition. αTubulin and Ponceau-S were used as an internal control, and anti-V5 antibody was used to detect expression and secretion of the constructs.

(C and D) Western blotting for biotinylated proteins (detected by Streptavidin-HRP) in hemolymph (C) and fat body (D). *LacZ*, *BirA*G3-ER*, *V5-TbID*, and *Mgat2-TbID* were expressed in fat body with *Gnmt^T2A-Gal4^*. Samples were collected in Non-Ablation (NA) condition. αTubulin and Ponceau-S were used as a loading control.

(E) Western blotting for biotinylated proteins (detected by Streptavidin-HRP) in the hemolymph ex vivo culture. LacZ, V5-TbID, and Mgat2-TbID were expressed in fat body with Gnmt^T2A-Gal4^ and maintained on normal food. Collected larval hemolymph in each condition was incubated for 1 hr in 50 μM Biotin medium in the presence or absence of supplemented 100 μM ATP. αTubulin and Ponceau-S were used as an internal control, and anti-V5 antibody was used to detect expression and secretion of the constructs.

(F and G) Western blotting for biotinylated proteins (detected by Streptavidin-HRP) in hemolymph (F) and whole larvae (G). *LacZ*, *V5-TbID*, and *Mgat2-TbID* were expressed in muscle with *Mef2^Gal4^*, in hemocyte with *Pxn^Gal4^*, and salivary gland with *Fkh^Gal4^*. Samples were collected in Non-Ablation (NA) condition. αTubulin and Ponceau-S were used as a loading control, and anti-V5 antibody was used to detect expression and secretion of the constructs.

**Figure S6**


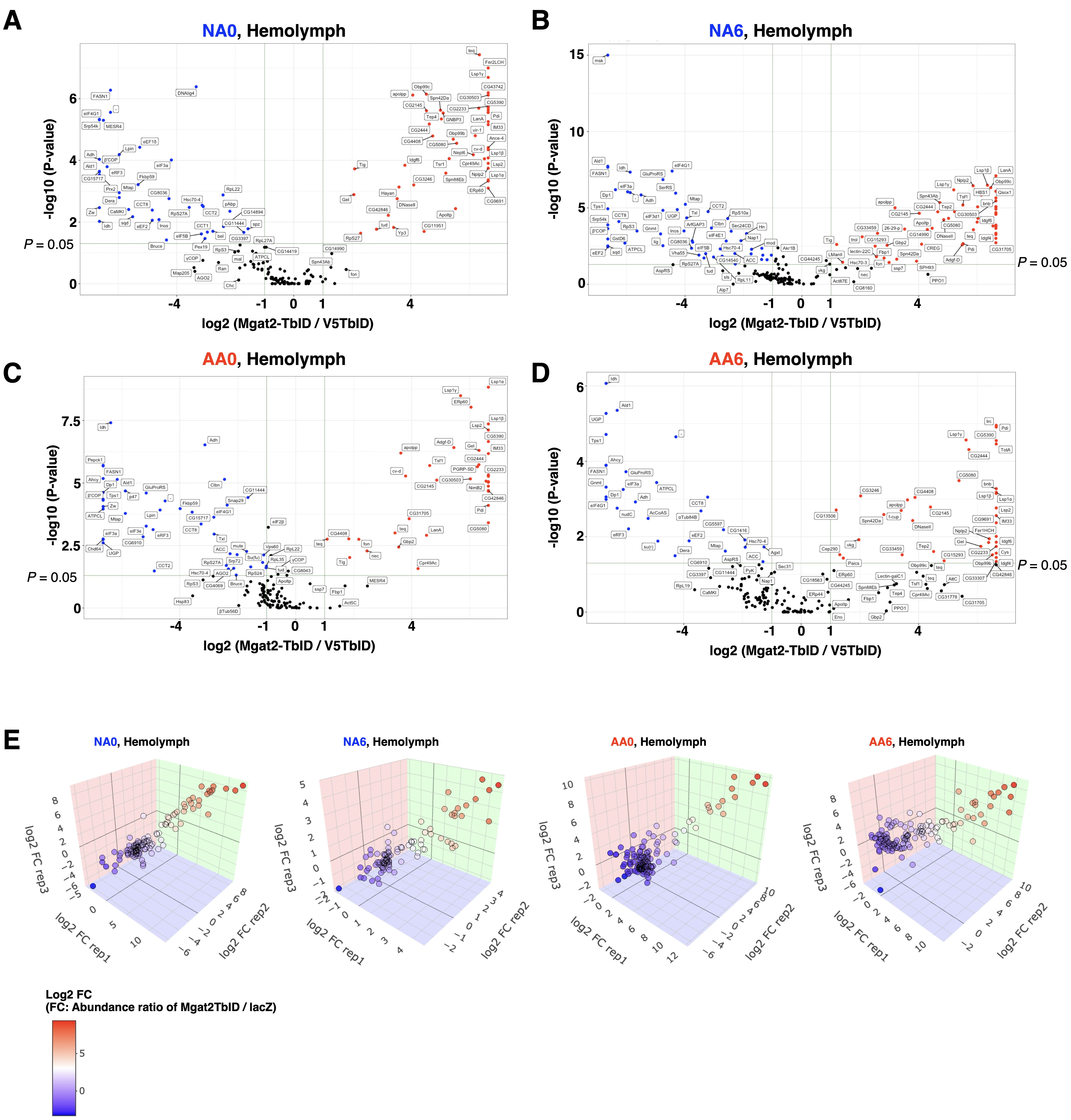


**Figure S6** **Volcano plots and replicate correlations of hemolymph proteomics data**

(A–D) Volcano plots of the hemolymph proteome comparing the abundance of Mgat2-TbID with V5-TbID at four time points: NA0 (A), NA6 (B), AA0 (C), and AA6 (D). Each point represents a quantified protein; the x-axis indicates the log2 abundance ratio (Mgat2-TbID/V5-TbID), and the y-axis indicates −log10(P). Each sample contained 15–25 larvae, with three biological replicates.

(E) Replicate correlation analysis of the hemolymph proteome comparing the abundance of Mgat2-TbID with lacZ at four time points (NA0, NA6, AA0, and AA6). Each point represents a quantified protein plotted by its log2 abundance ratio (Mgat2-TbID/lacZ) across three independent biological replicates (x-axis, replicate 1; y-axis, replicate 2; z-axis, replicate 3). Each sample contained 15–25 larvae.

**Figure S7**


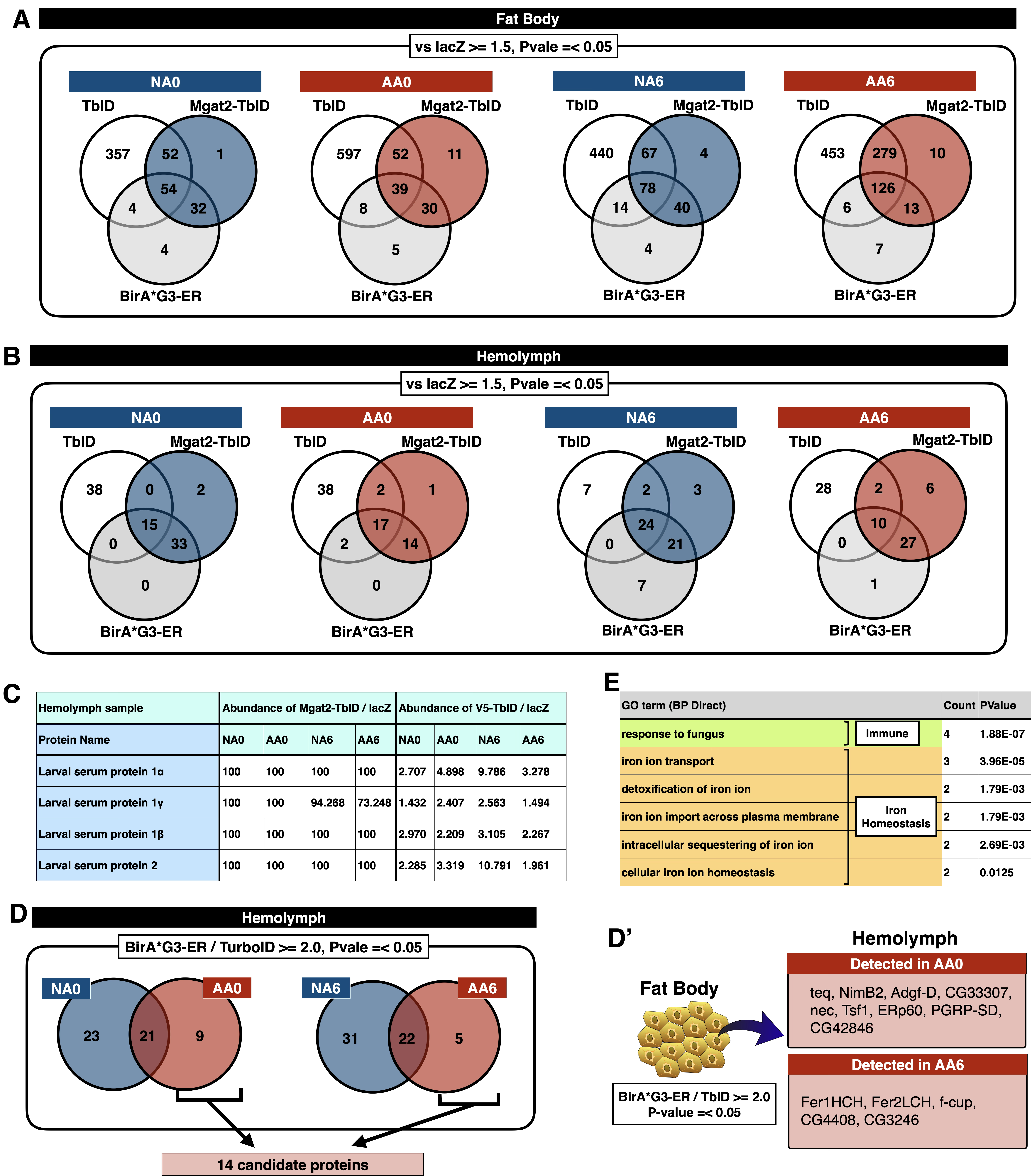


**Figure S7** **Proximal proteins labeled by Mgat2-TbID closely resemble those labeled by BirA*G3-ER**

(A and B) Analysis of biotinylated proteins in hemolymph (A) and fat body (B) at two time points. Proteins detected with each genotype at ≧1.5-fold higher abundance than *lacZ* and a p-value of ≤0.05 were extracted, and their numbers were listed.

(C) Labeled abundance of major larval secretory proteins (Lsp1α, Lsp1β, Lsp1γ, and Lsp2) in the hemolymph sample. Abundance ratio of Mgat2-TbID vs lacZ and TbID vs lacZ was used. In the analysis by Proteome Discoverer 2.2, ratio abundance data display a maximum value of 100, so values greater than or equal to 100 are displayed as 100.

(D and D’) Analysis of biotinylated proteins in the hemolymph at two time points. Under each condition, proteins detected with *BirA*G3-ER* at more than twice the amount when compared to *V5-TbID*, and with a p-value of 0.05 or less, were extracted. Among these proteins, 14 were specific to Ablation (D’). 15–25 larvae were used for a single sample, and biological replicates were n=3.

(E) GO analysis of 14 proteins in (D) with DAVID.

**References**

47. Kashio, S., and Miura, M. (2025) S-adenosylmethionine metabolism buffering is regulated by a decrease in glycine N-methyltransferase via the nuclear ubiquitin–proteasome system. *Proceedings of the National Academy of Sciences*. **122**, e2417821122

48. Grönke, S., Beller, M., Fellert, S., Ramakrishnan, H., Jäckle, H., and Kühnlein, R. P. (2003) Control of fat storage by a Drosophila PAT domain protein. *Current Biology*. **13**, 603–606

49. Kennerdell, J. R., and Carthew, R. W. (2000) Heritable gene silencing in Drosophila using double-stranded RNA. *Nat Biotechnol*. **18**, 896–898
